# Impact of precise preoperative vascular assessment and different dorsal pancreatic artery variant subtypes on pancreatic surgery-related bleeding

**DOI:** 10.1186/s12876-026-04687-8

**Published:** 2026-02-14

**Authors:** Jinshou Yang, Jiahao Xu, Ming Wang, Bohui Yin, Hanyang Yu, Chenjun Jiang, Qiang Xu, Yupei Zhao

**Affiliations:** 1 Department of General Surgery, Peking Union Medical College Hospital, Chinese Academy of Medical Science and Peking Union Medical College, Shuaifuyuan 1st, Dongcheng District, Beijing, 100730 China; 2 Department of Radiology, Peking Union Medical College Hospital, Chinese Academy of Medical Science and Peking Union Medical College, Beijing, 100730 China; 3 State Key Laboratory of Complex, Severe, and Rare Diseases, Peking Union Medical College Hospital, Chinese Academy of Medical Science and Peking Union Medical College, Beijing, 100730 China; 4 School of Basic Medical Sciences, Tsinghua Medicine, Tsinghua University, Beijing, China

**Keywords:** Pancreatic surgery, Dorsal pancreatic artery, Artery variation, Surgical bleeding

## Abstract

**Background:**

The variability of pancreatic vasculature, especially the dorsal pancreatic artery (DPA), increase surgical difficulty and may elevate the risk of intra- and postoperative bleeding. This study aimed to establish a precise preoperative vascular assessment protocol for pancreatic surgery, summarize DPA variant patterns, and evaluate their impact on pancreatic surgery-related bleeding.

**Methods:**

In this prospective study, 206 patients undergoing pancreatic surgery were included and evaluated preoperatively using computed tomography (CT) imaging and Preoperative Accurate Assessment Form for Pancreatic Vascular Variations (PAAF-PVV). 50 historical controls who underwent pancreatic surgery without PAAF-PVV were retrospectively included. DPA variants were systematically classified. The impact of PAAF-PVV-based vascular assessment and DPA variants on bleeding outcomes was analyzed.

**Results:**

Among patients who underwent precise preoperative vascular assessment for the pancreas (*n* = 148) versus those who did not (*n* = 32), no significant differences were observed in intraoperative blood loss, PPH incidence and postoperative hemoglobin decline (ΔHb). However, in the distal pancreatectomy group, the hemoglobin decline on POD2 differed significantly (ΔHb_POD2-POD1, -5.11 vs. -10.69 g/L, 95% CI 1.45–9.71, *P* = 0.010).

Then, DPA origins were classified into five types and no significant association was found with intraoperative blood loss or PPH incidence. However, type IIB DPA may increase the risk of early postoperative hemoglobin decline, whereas type IC DPA appeared to be associated with a lower risk, as reflected by ΔHb_POD2-POD1 values (-12.45 ± 11.605 vs. -1.15 ± 6.902 g/L, *P* = 0.046). DPA branching patterns were also documented. Patients with DPA head-side branch (HB) showed more postoperative hemoglobin decline than those without HB in distal pancreatic surgery, as reflected by ΔHb_POD3-POD1 values (-11.65 ± 6.434 vs. -7.45 ± 8.667 g/L, *P* = 0.049).

Interestingly, we also found that centro-inferior pancreatic vein (CIPV) drainage type was associated with ΔHb_POD3-POD1, with inferior mesenteric vein (IMV) drainage type linked to greater hemoglobin decline (-12.52±11.422 vs. -7.27±9.508 g/L, *P*=0.009). Besides, the minimally invasive surgical approach, distal pancreatic resection, and benign pancreatic disease appeared to be associated with fewer intra-operative blood loss.

**Conclusion:**

Variations in the pancreatic vasculature, including both arterial and venous systems, may influence surgery-related bleeding. Robust statistical evidence for bleeding reduction is not established in the overall study. Although the clinical outcome related measures presented in this study are merely associative and exploratory findings, a precise preoperative vascular assessment could help enhance anatomical understanding and optimize preoperative procedural planning, which is particularly valuable for surgeons during their learning phase.

**Supplementary Information:**

The online version contains supplementary material available at 10.1186/s12876-026-04687-8.

## Introduction

Pancreatic surgery-related hemorrhage includes intraoperative hemorrhage (IOH) and postoperative hemorrhage (or post-pancreatectomy hemorrhage, PPH), with PPH being the most severe postoperative complication. The incidence of PPH is approximately 2%-12%, and among those patients, the mortality rate is significantly increased, with Grade C PPH mortality as high as 34.2% [1]. Among all types of postoperative pancreatic hemorrhage, arterial bleeding is the most dangerous. Common sources of arterial bleeding include the superior/inferior pancreaticoduodenal arteries (S/I-PDA), the gastroduodenal artery (GDA), the jejunal artery (JA), the common hepatic artery (CHA), and the splenic artery (SpA) [2]. Effective bleeding control is a critical factor influencing patients’ mortality rates [3]. Variations in the pancreatic vasculature and the difficulty in handling them during operation are important causes of hemorrhage related to pancreaticoduodenectomy (PD) [4–6]. Mansour, S. et al. has shown that the IOH rate is 14.6% in the group with pancreatic vascular variations compared to the control group with 0% of IOH among patients that received PD [6]. Therefore, accurately assessing pancreatic vascular variations before surgery is very important.

In recent years, an increasing number of studies have reported on variations of the dorsal pancreatic artery (DPA) and their impact on surgical safety. DPA is a critical vessel supplying blood to the pancreas. Anatomically, DPA exhibits notable variability in its origin and branching patterns. It most commonly originates from the SpA (31–58%), followed by the superior mesenteric artery (SMA) (27–38%), the CHA (8–18%), and the celiac trunk/artery (CA) (10%)​ [5, 7]. Less frequently, it arises from arteries such as the inferior pancreaticoduodenal artery (IPDA) or the replaced right hepatic artery (rRHA) [7]. *Masahiro et al.* identified four main variants in DPA’s branches pattern: the superior branch (32%), the inferior branch (86%), the right branch (80%), and the accessory middle colic artery (12%) [8]. Actually, some DPA branches anastomoses with other arterial systems, including the posterior/anterior superior pancreaticoduodenal artery (P/A-SPDA), GDA, and the branches of SpA, complicating the task of defining the precise origin and branching patterns of the DPA. When DPA arises from the CHA or CA, the artery maybe easily overlooked by surgeons, potentially leading to inadvertent bleeding [9]. Research has demonstrated that early identification and ligation of the DPA during procedures such as pancreatoduodenectomy (PD) can significantly reduce intraoperative blood loss and shorten operative time [10]. However, it remains unclear whether specific arterial variants of DPA origin and branches have a substantial effect on pancreatic surgery-related bleeding including both IOH and PPH.

However, there is currently no unified and precise preoperative vascular assessment methods or tools. Contrast-enhanced computed tomography (ceCT) with three-dimensional reconstruction plays an indispensable role in preoperative planning for pancreatic surgeries, enabling the identification of the DPA in up to 92% of patients. This allows surgeons to assess the DPA’s origin, branching patterns, and potential anastomoses [8]. Thus, we have developed the “Preoperative Accurate Assessment Form for Pancreatic Vascular Variations (PAAF-PVV)” based on ceCT examination, which includes 20 pancreatic arteries and veins, involving hundreds of vascular variations (Supplementary_PAAF-PVV). We also conduct a comprehensive evaluation of the DPA’s origin and branching patterns in patients undergoing pancreatic surgeries, subsequently refining the classification method of DPA variants. Then, we investigated the impact of preoperative vascular evaluation by using PAAF-PVV and different DPA origin classifications and branches types on surgery-related bleeding.

## Methods

### Patient characteristics

We retrospectively included 50 patients at Peking Union Medical College Hospital (PUMCH) who were not evaluated by using PAAF-PVV, and excluded those who underwent pancreatic tumor enucleation (EN) and central pancreatectomy, ultimately enrolling 32 patients (Supplementary Fig. 1a). The prospective cohort included 206 patients who underwent pancreatic surgery at PUMCH between December 2022 and April 2024; excluding patients who underwent EN, central pancreatectomy, and duodenum preserving resection of head of pancreas (DPRHP), and patients whose DPA origin assessment was not completed, 148 eligible patients were enrolled (Supplementary Fig. 1b). The selected patients from prospective cohort, admitted for pancreatic diseases or duodenal masses, underwent routine preoperative examinations and thorough assessments of multiple pancreatic vascular variations including DPA by using PAAF-PVV. After excluding absolute surgical contraindications, they received surgical treatment, including PD, distal pancreatectomy (DP), or total pancreatectomy (TP). We categorized the surgical procedures into two major classes based on pancreatic head resection status: (1) Proximal pancreatic surgeries, defined as procedures involving resection of at least the pancreatic head; (2) Distal pancreatic surgeries, defined as procedures involving resection of the distal pancreas. The postoperative diet management for pancreatic surgery patients is highly standardized in our hospital: nil per os (NPO) on postoperative day 1 (POD1), 50 mL clear liquid on POD2, and 100 mL clear liquid on POD3. All patients receive intravenous fluids at 35 mL/kg and parenteral nutrition at 25 kcal/kg. All routine blood draws were performed at 7:00 AM. Two patients received transfusion therapy prior to 7am on POD2 (Supplementary Table 10).

The Institutional Ethical and Scientific Review Board of the Peking Union Medical College Hospital, Chinese Academy of Medical Science and Peking Union Medical College approved the study protocol (approval number: Scientific Research Quick Review Y1076, 2024). All procedures performed in studies were in accordance with the ethical standards of the institutional and national research committee and with the 1964 Helsinki declaration and its later amendments or comparable ethical standards. The clinical trial number is not applicable. Informed consent to participate in this study was obtained from all participants.

### Precise assessment procedure

The surgical team initially developed a ceCT based preoperative vascular assessment form for the pancreas based on published literature and clinical experience, which was refined iteratively through the clinical practice of more than 10 pancreatic surgeries to form the final version (Supplementary_PAAF-PVV). This assessment protocol represents a single-center expert-level operational framework pending external validation. The core vessels under assessment include 12 arteries and 15 veins, with the assessment dimensions covering vascular origin, course characteristics, number of branches, variant types, and intervascular positional relationships, among other key features.

For all patients in this study, the preoperative imaging of pancreatic vessels was first independently evaluated by the main operating surgeon, and subsequently reviewed and verified by two senior doctors from the surgical team for cross-checking and correction. The intraoperative vascular anatomical findings of the pancreas were collectively assessed by all doctors participating in the operation. All assessors received standardized and unified training, with clear clarification of the criteria for vascular identification, definitive definitions of assessment indicators, and specific rules for judging the matching degree of vascular findings. This standardized training regimen ensured a uniform workflow for pancreatic vascular assessment and guaranteed interobserver consistency in the evaluation process.

The congruence between preoperative imaging and intraoperative findings was categorized into the following scenarios (Supplementary Table 1): (1) Complete match: Preoperative CT assessment was entirely consistent with intraoperative observations. (2) Possible match: Preoperative CT assessment aligned with intraoperative observations but lacked full certainty, due to the entire course of the DPA was not fully exposed during surgery. (3) Mismatch: Preoperative CT assessment differed from intraoperative observations.

### 3D modeling

This study utilized the IQQA-3D system (EDDA Technology, Shanghai, China). Applying its patented four-dimensional image processing and analysis technology, the system separately characterizes the Hounsfield Unit (HU) values and anatomical features of different tissues and organs to generate a three-dimensional, fully quantifiable electronic organ map (image resolution: 512 × 512, pixel size: 0.48 × 0.48 to 0.98 × 0.98 mm). By converting the data into STL file format via the IQQA-3D system, the STL information for different organs, ducts, tissues, and tumors was used to create high-precision, transparent, and colored individualized physical models through full-color, one-piece 3D printing technology (material: photosensitive resin; process: stereolithography). The vascular precision meets the standard of 5th-order branches, with terminal vessel diameter “reaching the limit of CT slice resolution” providing sub-millimeter accuracy.

Eight patients were selected from the cohort of 206 to construct 3D virtual models using IQQA^®^-eQMR technology. Surgeons utilized 3D virtual reality (VR)/augmented reality (AR) glasses for a clearer assessment of DPA vascular variations. Five cases of them were selected for 3D printing.

### Clinical outcome indicators

The analysis of surgery-related bleeding was mainly based on the following indicators:

1. Primary outcomes: Intraoperative blood loss (mL), recorded during surgery; Occurrence of post‑pancreatectomy hemorrhage (PPH), defined according to ISGPS criteria.
2. Secondary outcomes (exploratory signals): Decline in hemoglobin on postoperative day 1 relative to preoperative levels (ΔHb_POD1-Pre, g/L); Postoperative hemoglobin trend, measured as the change in hemoglobin on postoperative days 2 and 3 relative to day 1 (ΔHb_POD2-POD1, ΔHb_POD3-POD1, g/L).

Intraoperative blood loss directly reflects surgical bleeding, while ΔHb_POD1-Pre provides an objective laboratory correlate of perioperative blood loss.

### Statistics

Normality is evaluated via two complementary approaches: the Shapiro-Wilk test is preferred for small-to-moderate samples, while the Kolmogorov-Smirnov test is more suitable for large samples. Dependent variables (intraoperative blood loss and changes in postoperative hemoglobin) were analyzed using analysis of variance (ANOVA) or Kruskal-Wallis tests for categorical variables and linear regression for continuous variables. Small sample groups were analyzed by non-parametric tests: replacing independent-samples t-tests with the Mann-Whitney U test, one-way ANOVA with the Kruskal-Wallis H test, and paired t-tests with the Wilcoxon signed-rank test, as these methods require no normality assumption. PPH grades were analyzed using chi-square or Fisher’s exact tests for categorical variables and ANOVA or Kruskal-Wallis tests for continuous variables. DPA origin variation analysis was performed twice: (1) Analysis of broad DPA types (I, II, III, absent type) and surgery-related bleeding; (2) A more detailed analysis of subtypes (IA, IB, IC, IIA, IIB, III, absent type) and surgery-related bleeding. While the second analysis provided greater specificity, the smaller sample size increased random error. All statistical analyses were conducted using SPSS software.

## Results

### DPA origin types

**Fig. 1:**
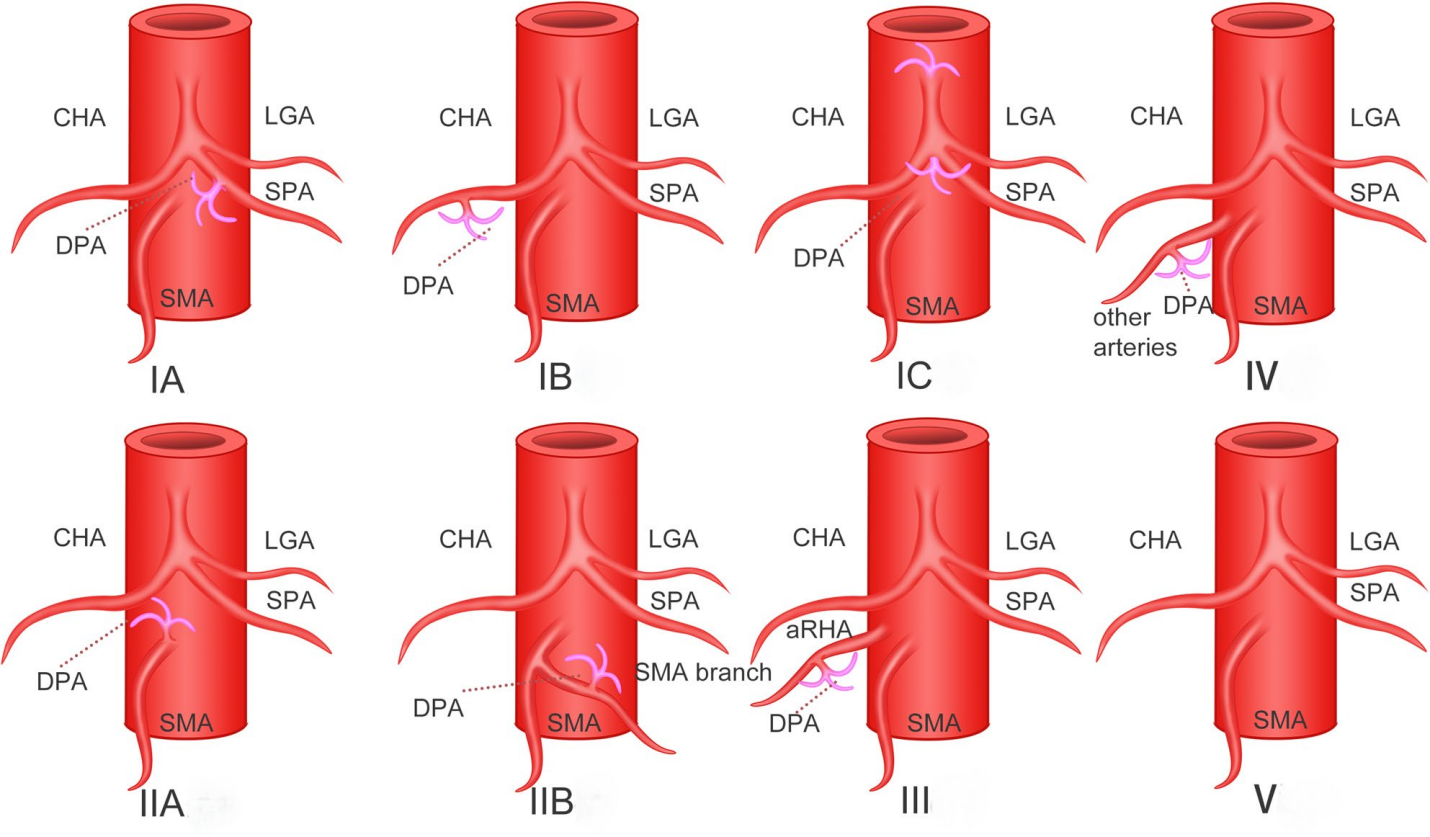
Subtypes of different dorsal pancreatic artery (DPA) origins. Type IA originates from splenic artery (SpA), 22.9%; Type IB originates from common hepatic artery (CHA), 13.3%; Type IC originates from celiac trunk/artery (CA) or bifurcation, 10.2%; Type IIA originates from superior mesenteric artery (SMA) trunk, 24.7%; Type IIB originates from branches of SMA including middle colic artery (MCA) (*n*=6), inferior pancreaticoduodenal artery (IPDA) (*n*=5), the first jejunal artery (JA1) (*n*=5), JA2 (*n*=1), left colic artery (LCA) (*n*=2), left gastric artery (LGA) (*n*=1), totally accounting for 12%. Type III originates from the aberrant right hepatic artery (aRHA), 4.2%; Type IV originates from other arterial systems rarely, such as the abdominal aorta or phrenic arteries, 0.6%; Type V refers to not exist, 12%

Through precise preoperative vascular assessment, we divided DPA into five types based on origin location (Table 1). The overall prevalence of DPA was approximately 88%, with the majority originating from the CA system (46.4%) and the SMA system (36.7%), and 4.2% originating from the aRHA. In order to better understand the origins of the DPA from an anatomical perspective, we further subdivided these origins into more subtypes (Figs. 1). Type IA originates from SpA; Type IB originates from CHA; Type IC originates from CA trunk or bifurcation; Type IIA originates from SMA trunk; Type IIB originates from branches of SMA including middle colic artery (MCA) × 6, IPDA × 5, JA1 × 5, JA2 × 1, left colic artery (LCA) × 2, left gastric artery (LGA) × 1. The majority of DPA origins were from the SpA (22.9%) and SMA trunk (24.7%), followed by the CHA (13.3%), SMA-arising branch vessels (12%), and the trunk or bifurcation of the CA (10.2%).

**Table 1:** The DPA origin types based on the preoperative precise vascular assessment

Types	*N*	Ratio	Definition
I	77	46.4%	DPA originates from the CA system.
IA	38	22.9%	DPA originates from SpA.
IB	22	13.3%	DPA originates from CHA.
IC	17	10.2%	DPA originates from CA trunk or bifurcation.
II	61	36.7%	DPA originates from the SMA system.
IIA	41	24.7%	DPA originates from SMA trunk.
IIB	20	12.0%	DPA originates from branches of SMA.
III	7	4.2%	DPA originates from aRHA.
IV	1	0.6%	DPA originates from other arteries (such as aorta abdominalis, phrenic artery).
V	20	12.0%	No DPA was found.

A comprehensive preoperative imaging assessment is strongly recommended. The match rates between preoperative imaging assessment and intraoperative observations for DPA origin were approximately 74.6% for fully matched, 35.6% for potentially matched, and 5.1% for mismatched (Supplementary Table 1). “Potentially Matched (uncertain)” was usually contributed to inadequate intraoperative exposure or unnecessary intraoperative exposure. “Mismatched” was mostly due to preoperative misjudgment, or the DPA was not seen before surgery, but was seen intraoperatively. The proportions of DPA origin types observed during surgery were nearly identical to the preoperative imaging results, except for Type IC, which showed obvious difference (Supplementary Table 2). Preoperative imaging evaluation of DPA origin at the CA bifurcation (Type IC) is technically challenging and prone to inaccuracies.

### DPA branches distribution

**Fig. 2:**
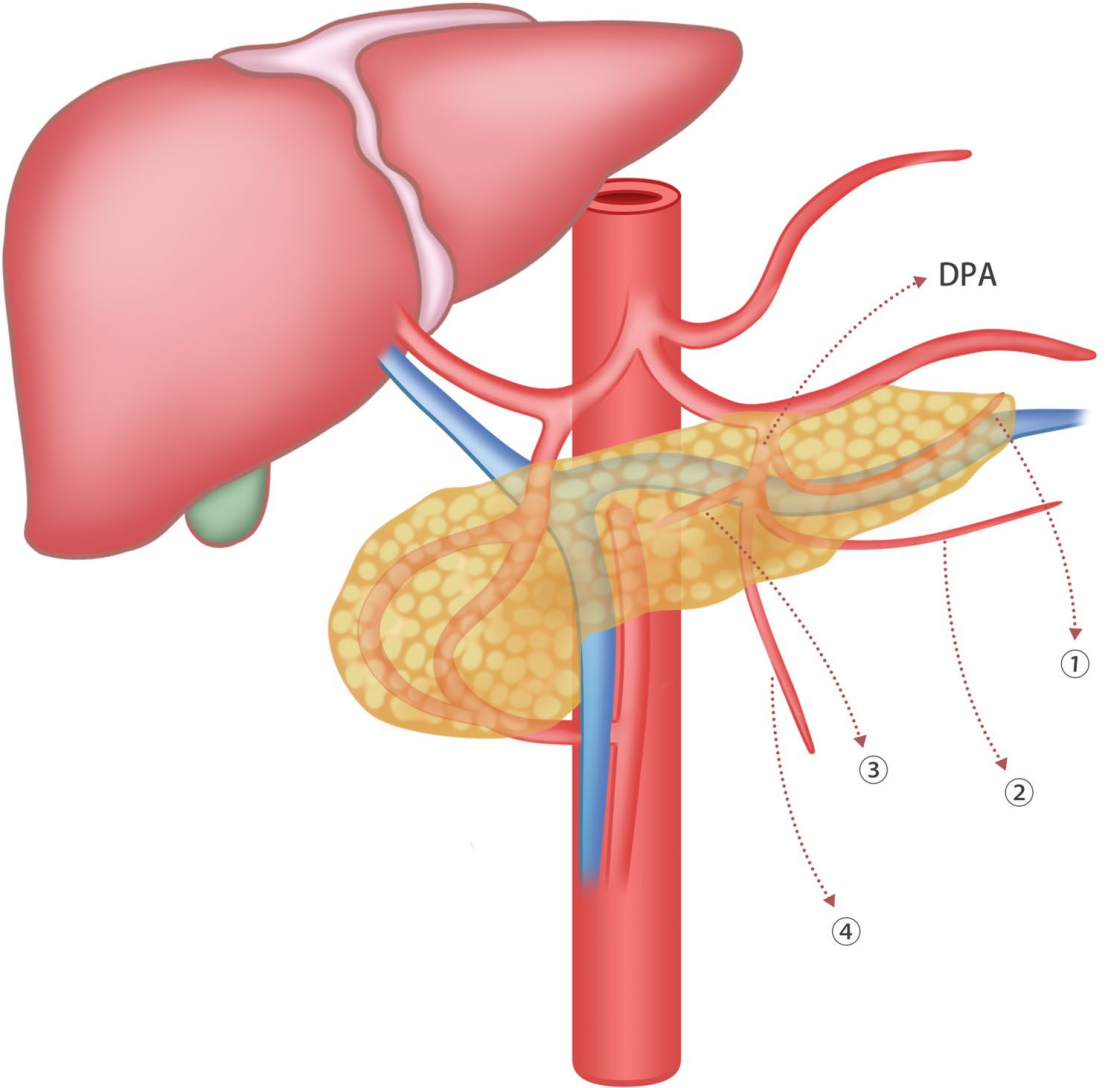
Subtypes of dorsal pancreatic artery (DPA) branches. ① Head-side branch (HB) that courses into the pancreas cephalad to the splenic vein (SpV), representing the superior branch, 73%; ② Foot-side branch (FB) that courses into the pancreas caudad to SpV, representing the inferior branch, 90.8%; ③Uncinate process branch (UB) that travels towards the uncinate process of the pancreas, representing the right branch, 75.9%; ④ Extra branches (ExB), including inferior pancreaticoduodenal artery branch (IPDB), jejunal artery branch (JB), or colic artery branch (CB), totally accounting for 14.2%

We parsimoniously classified DPA branches into four types based on published studies and our clinical experience: Head-side branch (HB) that courses into the pancreas cephalad to the splenic vein (SpV), representing the superior branch; Foot-side branch (FB) that courses into the pancreas caudad to SpV, representing the inferior branch; Uncinate process branch (UB) that travels towards the uncinate process of the pancreas, representing the right branch; Extra branches (ExB), including IPDA branch (IPDB), jejunal artery branch (JB), or colic artery branch (CB). We found that the FB was the most common branch (90.8%), followed by UB (75.9%), HB (73%), and ExB (14.2%) (Table 2; Fig. 2). In fact, the IPDA also courses along the uncinate process; it was classified as an extra branch because the course of the IPDB is closer to the caudal side than the UB.

**Table 2:** The DPA branches distribution based on preoperative precise vascular assessment

Types	*N*	Ratio
HB	103	73.0%
FB	128	90.8%
UB	107	75.9%
ExB	20	14.2%
IPDB	11	7.8%
JB	2	1.4%
CB	7	5.0%

The match rates between preoperative imaging assessment and intraoperative observations for DPA branches were 46.1% for fully matched, 47.1% for potentially matched, and 6.9% for mismatched (Supplementary Table 3). While the proportion of “fully matched” was relatively low, it is noteworthy that the identification of specific branches achieved strong concordance between preoperative imaging evaluation and intraoperative observation. (Supplementary Table 4).

### 3D models construction

We selected representative cases of different DPA variation types and displayed 3D anatomical models of the pancreas, tumors, blood vessels, and surrounding organs using virtual reality (VR) technology. These models were further reproduced through 3D printing technology (Supplementary Fig. 3–7), providing a clearer understanding of the spatial relationships among these anatomical structures. Through 3D printing, we were able to offer detailed educational explanations to young surgeons about DPA origin variations, branching patterns, and the importance of preoperative evaluations. Some hospitals have explored the application of novel materials in 3D printing to enhance the clinical utility of surgical planning. Despite the considerable expenses associated with current 3D printing, its prospective clinical benefits are profound.

### Evaluation of the clinical application value of PAAF-PVV

**Fig. 3:**
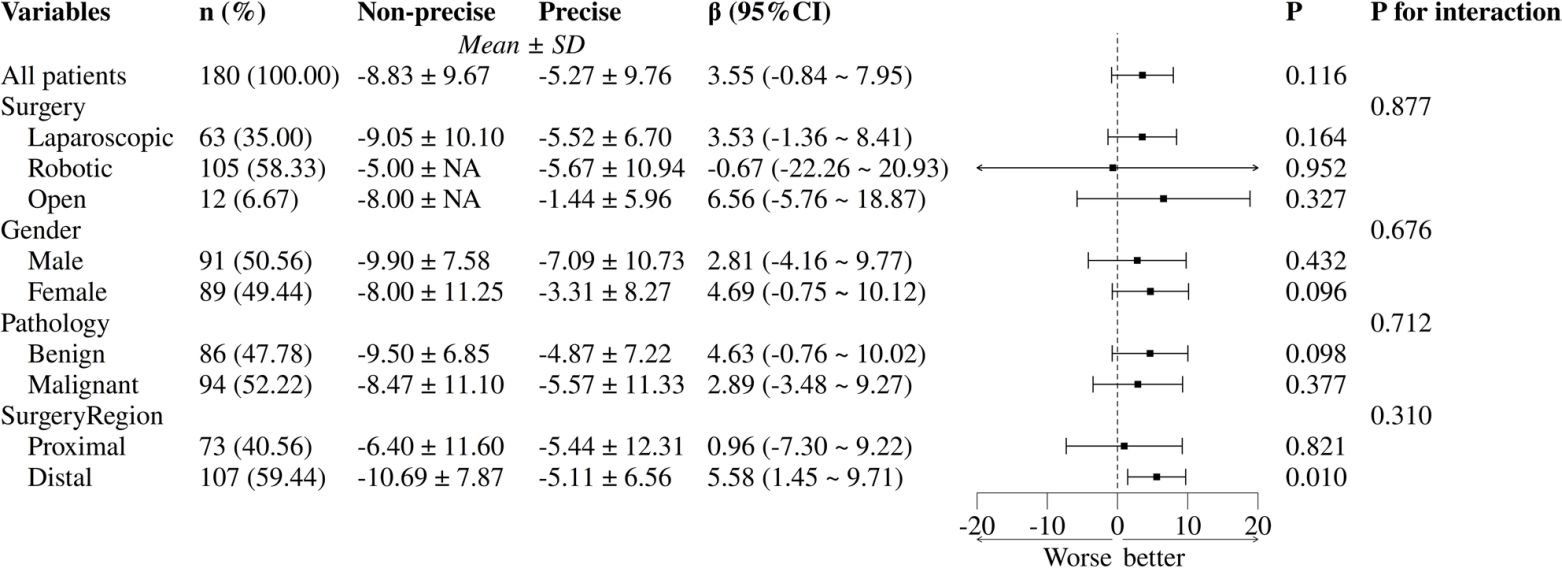
Subgroup analysis of ΔHb_POD2-POD1 between non-precise assessment and precise preoperative vascular assessment. Non-Precise: patients receiving non-precise preoperative vascular assessment (*n* = 32); Precise: patients receiving precise preoperative vascular assessment (*n* = 148). In sub-group of distal pancreatic surgery, patients receiving precise assessment showed lower hemoglobin decline in POD2 than patients receiving non-precise assessment (-5.11 ± 6.56 vs. -10.69 ± 7.87, *P* = 0.010). The analysis was not adjusted for covariates. This forest plot presents exploratory subgroup analyses, indicating the preliminary nature of the findings

We retrospectively included 32 patients who did not undergo precise vascular assessment of the pancreas before surgery, and prospectively included 148 patients who underwent precise vascular assessment of the pancreas before surgery using PAAF-PVV. Analysis results showed that between the two groups, there was no significant difference in the intraoperative bleeding and PPH incidence (Table 3). Next, we performed exploratory subgroup analyses of postoperative hemoglobin changes. The results suggested that ΔHb_POD2-POD1 showed significant difference in distal pancreatic surgeries (Fig. 3; Supplementary Fig. 2a-c). Although statistical significance was not achieved after adjusting for covariates (Supplementary Fig. 2d-g), the findings revealed that precise preoperative vascular assessment facilitates preoperative pancreatic surgical planning.

**Table 3:** Comparative analysis of clinical characteristics between non-precise vascular assessment and precise vascular assessment

		Non-precise assessment	Precise assessment	*P*
Gender	Male	14 (43.7%)	77 (52%)	0.396
	Female	18 (56.3%)	71 (48%)	
Age	years	55.97 ± 16.039	57.08 ± 13.638	0.686
Surgical method	Open	1 (3.1%)	11 (7.5%)	<0.001
	Laparoscopic	28 (87.5%)	35 (23.6%)	
	Da Vinci robot-assisted	3 (9.4%)	102 (68.9%)	
Resection area	Proximal pancreatic surgery	13 (40.6%)	60 (40.5%)	0.993
	Distal pancreatic surgery	19 (59.4%)	88 (59.5%)	
Pathology type	Benign	13 (40.6%)	73 (49.3%)	0.372
	Malignant	19 (59.4%)	75 (50.7%)	
Intraoperative bleeding	Blood loss (mL)	365.94 ± 374.991	306.01 ± 386.959	0.426
	ΔHb_POD1-Pre (g/L)	-12.25 ± 12.994	-10.04 ± 12.808	0.379
PPH	No	30 (93.8%)	134 (90.5%)	0.741
	Yes	2 (6.2%)	14 (9.5%)	
Postoperative hemoglobin change	ΔHb_POD2-POD1 (g/L)	-8.83 ± 9.670	-5.27 ± 9.763	0.116
	ΔHb_POD3-POD1 (g/L)	-13.19 ± 8.510	-10.26 ± 10.556	0.146
Postoperative transfusion	No	32 (100%)	142 (95.9%)	0.593
	Yes	0 (0%)	6 (4.1%)	
Postoperative intervention	No	31 (96.9%)	141 (95.3%)	0.690
	Yes	1 (3.1%)	7 (4.7%)	
Length of stay	days	13.53 ± 9.752	13.66 ± 9.497	0.947

### Analysis of surgery-related bleeding factors

#### Intraoperative blood loss

Surgical approach, resection area, pathological type, and postoperative length of stay were correlated with intraoperative blood loss, while DPA variant types show no significant association (Table 4). However, in the group undergoing proximal pancreatic surgery, DPA origin type was closely correlated with intraoperative blood loss (Supplementary Table 5a). The highest intraoperative blood loss was observed in patients with type IC DPA. However, the results of the multiple comparison analysis after adjusted by the Bonferroni correction did not show statistical significance (Supplementary Table 5b).

**Table 4:** The analysis of PPH and intraoperative bleeding-related factors

DPA variation		PPH			Intraoperative bleeding (mL)			ΔHb (POD1-Pre) (g/L)		
		No	Yes	*P*	*N*	Mean ± SD	*P*	*N*	Mean ± SD	*P*
Gender	Female	66 (49.3%)	5 (35.7%)	0.335	71	290.56 ± 364.997	0.642	71	-9.56 ± 12.549	0.647
	Male	68(50.7%)	9 (64.3%)		77	320.26 ± 408.031		76	-10.54 ± 13.186	
Age	years	56.72 ± 13.506	60.57 ± 14.919	0.316	*R* = 0.173,*P* = 0.035			*R* = 0.050,*P* = 0.550		
Surgical method	Open	9 (6.7%)	2 (14.3%)	0.485	11	1018.18 ± 632.168	<0.001^a^	11	-20.18 ± 21.127	0.333^a^
	LAP	31 (23.1%)	4 (28.6%)		35	256.29 ± 324.002		34	-10.18 ± 12.182	
	Robotic	94 (70.2%)	8 (57.1%)		102	246.27 ± 288.667		102	-8.94 ± 11.554	
Resection area	Proximal	47 (35.1%)	12 (85.7%)	<0.001	59	494.24 ± 498.478	<0.001	59	-12.44 ± 15.678	0.091
	Distal	87 (64.9%)	2 (14.3%)		89	181.24 ± 216.469		88	-8.48 ± 10.332	
Pathology type	Benign	69 (51.5%)	4 (28.6%)	0.103	73	240.82 ± 317.778	0.043	72	-11.49 ± 12.312	0.191
	Malignant	65 (48.5%)	10 (71.4%)		75	369.47 ± 436.949		75	-8.71 ± 13.282	
Origin type	IA	28 (20.9%)	4 (28.6%)	0.482	32	399.69 ± 466.307	0.185^a^	32	-13.09 ± 12.172	0.075
	IB	18 (13.4%)	2 (14.3%)		20	146.50 ± 104.291		20	-6.10 ± 15.269	
	IC	11 (8.2%)	3 (21.4%)		14	413.57 ± 658.852		14	-17.07 ± 14.668	
	IIA	36 (26.9%)	2 (14.3%)		38	282.89 ± 282.143		38	-8.66 ± 10.888	
	IIB	18 (13.4%)	0 (0%)		18	186.67 ± 177.333		17	-4.82 ± 8.049	
	III	7 (5.2%)	1 (7.1%)		8	366.25 ± 301.848		8	-10.87 ± 9.935	
	V	16 (11.9%)	2 (14.3%)		18	374.44 ± 482.728		18	-11.22 ± 15.902	
Branch number	No	4 (3.4%)	0 (0%)	0.897	4	400 ± 601.387	0.566	4	-13.50 ± 8.963	0.488
	1	12 (10.2%)	1 (8.3%)		13	315.38 ± 430.786		13	-11.69 ± 12.439	
	2	29 (24.6%)	2 (16.7%)		31	200.32 ± 160.966		31	-6.74 ± 9.14	
	3	63 (53.4%)	8 (66.7%)		71	330.28 ± 421.924		70	-10.24 ± 13.153	
	≥ 4	10 (8.5%)	1 (8.3%)		11	290.00 ± 314.261		11	-13.27 ± 16.42	
FB	No	13 (11.0%)	1 (8.3%)	0.775	14	292.14 ± 334.300	0.963	14	-9.50 ± 10.450	0.897
	Yes	105 (89.0%)	11 (91.7%)		116	297.07 ± 378.794		115	-9.96 ± 12.687	
HB	No	32 (27.1%)	2 (16.7%)	0.731	34	290.59 ± 458.171	0.914	34	-11.53 ± 10.715	0.377
	Yes	86 (72.9%)	10 (83.3%)		96	298.65 ± 340.664		95	-9.33 ± 12.991	
UB	No	28 (23.7%)	3 (25.0%)	0.922	31	279.68 ± 409.320	0.789	31	-8.71 ± 12.592	0.545
	Yes	90 (76.3%)	9 (75.0%)		99	301.82 ± 363.038		98	-10.29 ± 12.418	
ExB	No	105 (89.0%)	9 (75.0%)	0.168	114	267.81 ± 283.714	0.227	113	-9.29 ± 11.324	0.308
	Yes	13 (11.0%)	3 (25.0%)		16	501.25 ± 734.555		16	-14.25 ± 18.379	
IPDV drainage	Type I	29 (64.4%)	8 (72.7%)	0.732	37	498.65 ± 485.324	0.086	37	-12.46 ± 16.015	0.760
	Non-Type I	16 (35.6%)	3 (27.3%)		19	329.47 ± 237.965		19	-11.11 ± 14.817	
CIPV drainage	IMV	59 (51.8%)	5 (45.5%)	0.522	64	294.69 ± 352.857	0.512	63	-10.63 ± 11.77	0.359
	SMV	17 (14.9%)	0 (0%)		17	186.47 ± 196.053		17	-8.47 ± 10.835	
	SpV	8 (7.0%)	1 (9.1%)		9	423.33 ± 488.8		9	-14.11 ± 12.888	
	Colonic vein or JVT	20 (17.5%)	3 (27.3%)		23	250.00 ± 249.272		23	-10.87 ± 13.329	
	Dual drainage pattern	10 (8.8%)	2 (18.2%)		12	320.00 ± 457.900		12	-4.08 ± 13.440	
CIPV drainage	IMV	66 (57.9%)	6 (54.5%)	0.830	72	281.53 ± 337.546	0.942	71	-9.38 ± 12.066	0.515
	Non-IMV	48 (42.1%)	5 (45.5%)		53	286.04 ± 349.160		53	-10.83 ± 12.474	
Postoperative fistula	No	20 (14.9%)	0 (0%)	0.099	20	387.00 ± 348.668	0.604	20	-9.35 ± 16.554	0.958
	Biochemical	55 (41.0%)	4 (28.6%)		59	296.27 ± 384.967		58	-10.03 ± 10.601	
	B/C	59 (44.1%)	10 (71.4%)		69	290.87 ± 401.346		69	-10.30 ± 13.543	
Postoperative infection	No	121 (90.3%)	9 (64.3%)	0.015	130	283.08 ± 326.726	0.257	129	-9.72 ± 12.169	0.507
	Yes	13 (9.7%)	5 (35.7%)		18	471.67 ± 672.058		18	-12.56 ± 17.181	
Postoperative transfusion	No	134 (100%)	8 (57.1%)	<0.001	142	268.24 ± 303.566	0.051	141	-9.11 ± 12.114	<0.001
	Yes	0 (0%)	6 (42.9%)		6	1200 ± 896.660		6	-32.50 ± 9.006	
Postoperative intervention	No	134 (100%)	7 (50%)	<0.001	141	289.57 ± 342.945	0.342	140	-9.61 ± 12.228	0.282
	Yes	0 (0%)	7 (50%)		7	637.14 ± 888.833		7	-19.14 ± 21.248	
Length of stay	days	12.18 ± 6.071	27.79 ± 20.135	0.013	*R* = 0.279, ***P***<0.001			*R*=-0.224, ***P*** = 0.006		

a When the variance is uneven, a non-parametric test (Kruskal-Wallis test) is used

#### Post-pancreatectomy hemorrhage (PPH)

The incidence of PPH was significantly higher in proximal pancreatic surgeries than in distal pancreatic surgeries (Table 4). However, no correlation was observed between the types of DPA variants and PPH, whether in the entire cohort, in patients undergoing proximal pancreatic surgery, or in those undergoing distal pancreatic surgery (Supplementary Table 5a and 7).

### Exploratory analysis

#### Postoperative hemoglobin decline

Sex and DPA origin type were correlated with ΔHb_POD2-POD1 (Table 5). Patients with type IIB DPA exhibited the greatest hemoglobin decline on POD2, whereas those with type IC DPA showed the least - a pattern opposite to that observed for intraoperative blood loss. In the proximal pancreatic surgery group, sex remained closely associated with ΔHb_POD2-POD1 (Supplementary Table 6); Although type IIB patients showed the largest hemoglobin decline on POD2, the difference was not statistically significant, possibly due to the limited sample size. Additionally, in the distal pancreatic surgery group, ΔHb_POD3-POD1 was significantly greater in patients with a DPA head-side branch (HB) than in those without HB (Supplementary Table 8).

**Table 5:** The analysis of postoperative hemoglobin decline-related factors

DPA variation		ΔHb (POD2-POD1) (g/L)			ΔHb (POD3-POD1) (g/L)		
		*N*	Mean ± SD	*P*	*N*	Mean ± SD	*P*
Gender	Female	51	-3.31 ± 8.267	0.046	68	-8.75 ± 9.653	0.086
	Male	55	-7.09 ± 10.728		69	-11.86 ± 11.271	
Age	years	*R* = 0.085, *P* = 0.387			*R* = 0.160,*P* = 0.062		
Surgical method	Open	9	-1.44 ± 5.961	0.473	11	-9.55 ± 10.958	0.383
	LAP	25	-5.52 ± 6.703		33	-12.55 ± 13.271	
	Robotic	72	-5.67 ± 10.943		93	-9.61 ± 9.417	
Resection area	Proximal	52	-5.44 ± 12.310	0.864	52	-9.75 ± 14.470	0.675
	Distal	54	-5.11 ± 6.555		85	-10.66 ± 7.325	
Pathology type	Benign	45	-4.87 ± 7.219	0.714	71	-10.66 ± 7.724	0.696
	Malignant	61	-5.57 ± 11.331		66	-9.94 ± 13.019	
Origin type	IA	23	-7.70 ± 13.699	0.046	28	-11.43 ± 10.813	0.289
	IB	16	-5.81 ± 7.476		19	-12.11 ± 9.327	
	IC	13	-1.15 ± 6.902		13	-8.69 ± 11.375	
	IIA	23	-1.91 ± 7.769		35	-7.37 ± 8.433	
	IIB	11	-12.45 ± 11.605		16	-9.94 ± 7.280	
	III	6	-4.83 ± 3.061		8	-17.25 ± 22.192	
	V	14	-4.57 ± 6.525		18	-10.83 ± 9.237	
Branch number	No	4	-5.00 ± 5.354	0.985	3	-3.67 ± 9.292	0.220
	1	8	-5.12 ± 17.266		12	-6.83 ± 11.134	
	2	18	-6.33 ± 7.452		29	-8.59 ± 7.258	
	3	52	-5.40 ± 10.193		65	-12.26 ± 11.564	
	≥ 4	10	-3.90 ± 10.503		10	-7.90 ± 12.982	
FB	No	11	-7.00 ± 6.325	0.577	12	-8.33 ± 7.215	0.522
	Yes	81	-5.16 ± 10.615		107	-10.45 ± 11.131	
HB	No	24	-5.17 ± 11.228	0.906	32	-7.53 ± 9.249	0.098
	Yes	68	-5.46 ± 9.885		87	-11.23 ± 11.198	
UB	No	20	-4.75 ± 11.452	0.756	28	-7.93 ± 10.015	0.180
	Yes	72	-5.56 ± 9.891		91	-10.95 ± 10.981	
ExB	No	76	-5.66 ± 10.318	0.572	104	-10.29 ± 10.465	0.888
	Yes	16	-4.06 ± 9.760		15	-9.87 ± 13.293	
IPDV drainage	Type I	32	-7.12 ± 13.807	0.444	32	-10.34 ± 16.754	0.946
	Non-Type I	16	-4.12 ± 9.999		17	-10.65 ± 10.062	
CIPV drainage	IMV	42	-5.50 ± 6.029	0.289^a^	60	-12.10 ± 11.481	0.012
	SMV	13	-5.15 ± 6.606		17	-7.29 ± 6.488	
	SpV	5	-9.60 ± 17.126		9	-7.22 ± 8.614	
	Colonic vein or JVT	20	-0.25 ± 10.156		22	-5.41 ± 11.061	
	Dual drainage pattern	8	-11.75 ± 18.881		10	-17.60 ± 9.559	
CIPV drainage	IMV	48	-6.71 ± 9.381	0.087	67	-12.52 ± 11.422	0.009
	Non-IMV	40	-3.07 ± 10.316		51	-7.27 ± 9.508	
Postoperative fistula	No	16	-5.06 ± 8.737	0.995	18	-5.56 ± 9.031	0.021
	Biochemical	40	-5.27 ± 11.218		53	-9.02 ± 9.914	
	B/C	50	-5.34 ± 8.984		66	-12.65 ± 10.994	
Postoperative infection	No	90	-5.22 ± 10.117	0.899	121	-10.03 ± 10.584	0.395
	Yes	16	-5.56 ± 7.737		16	-12.44 ± 10.608	
Postoperative transfusion	No	100	-5.14 ± 8.024	0.838	131	-9.89 ± 8.393	0.511
	Yes	6	-7.50 ± 26.846		6	-19.50 ± 33.243	
Postoperative intervention	No	99	-5.73 ± 9.790	0.072	131	-9.78 ± 8.954	0.335
	Yes	7	1.14 ± 7.151		6	-22.00 ± 28.043	
Length of stay	days	*R* = 0.109, *P* = 0.268			*R*=-0.054, *P* = 0.528		

a When the variance is uneven, a non-parametric test (Kruskal-Wallis test) is used

#### Others

**Fig. 4:**
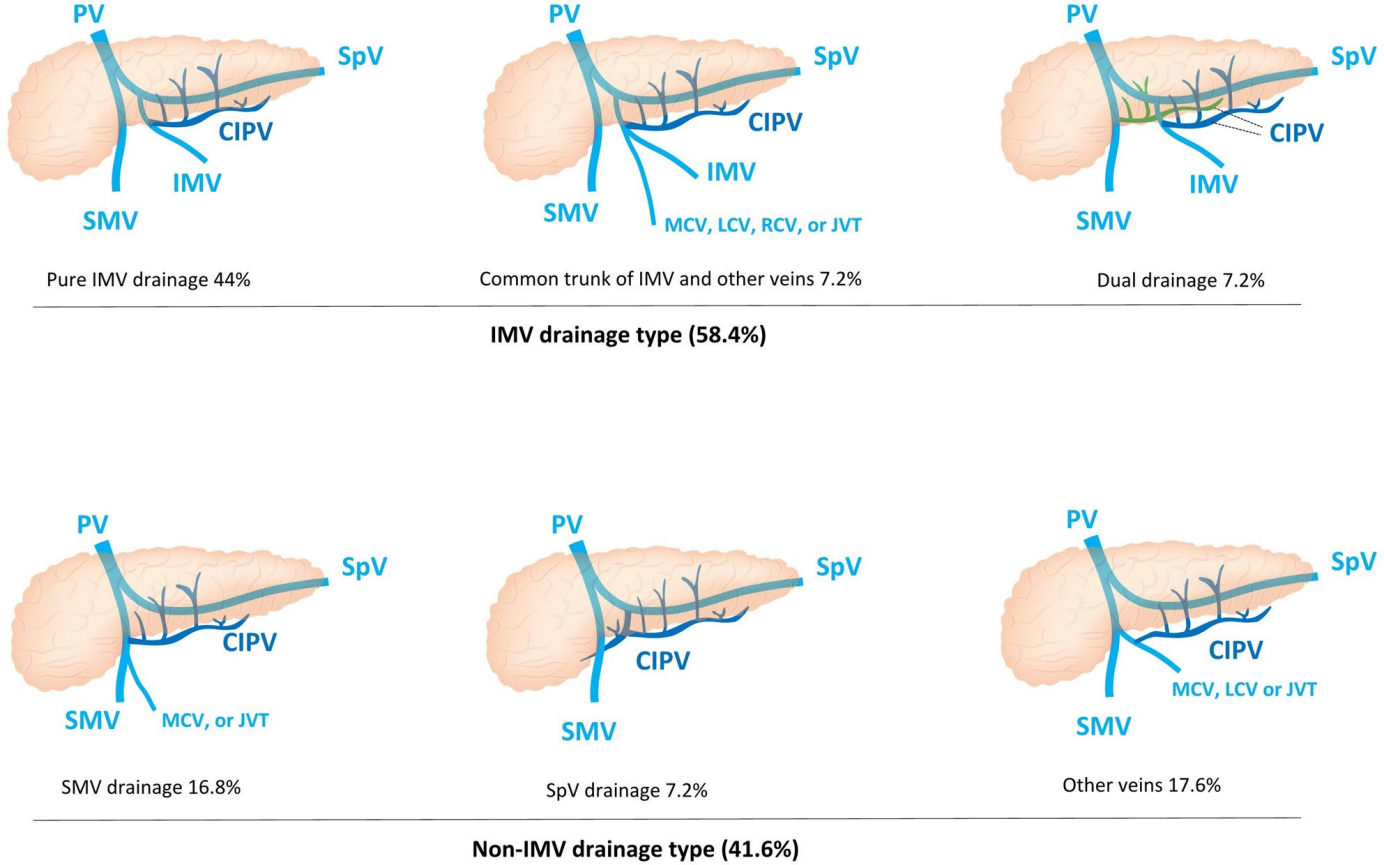
Centro-inferior pancreatic vein (CIPV) drainage patterns. ① IMV drainage: Pure IMV drainage accounts for 44%. Common trunk of IMV and other veins (MCV 2.4%, LCV 2.4%, RCV 0.8%, JVT 1.6%) accounts for 7.2%. Dual drainage including IMV accounts for 7.2% (IMV + SMV 3.2%, IMV + SpV 1.6%, IMV + MCV 2.4%). ② Non-IMV drainage: SMV drainage including pure IMV drainage type (13.6%) and the type of drainage into the common trunk of SMV and JVT (1.6%) or MCV (1.6%). Other veins including MCV (12.8%), LCV (2.4%) and JVT (3.6%). Abbreviations: CIPV centro-inferior pancreatic vein; IMV inferior mesenteric vein; JVT jejunal venous trunk; LCV left colic vein; MCV middle colic vein; RCV right colic vein; SMV superior mesenteric vein; SpV splenic vein

In this study, both intraoperative blood loss and PPH were correlated with postoperative hospital stay, whereas postoperative ΔHb showed no such correlation. We also examined the influence of inferior pancreaticoduodenal vein (IPDV) and centro-inferior pancreatic vein (CIPV) drainage patterns (Fig. 4) on surgery-related bleeding. Notably, CIPV drainage type was closely associated with ΔHb_POD3-POD1 (Table 5). Patients with IMV-type drainage had a greater hemoglobin decline by POD3, especially in those undergoing pancreatic head resection or those with dual drainage pattern (Supplementary Table 6). In a multivariable analysis incorporating DPA origin type, IPDV drainage type, and CIPV drainage type, CIPV drainage type remained associated with ΔHb_POD3-POD1 (Supplementary Table 9a). Meanwhile, we performed robust multivariable adjustment with more covariates included in the model, but statistical significance was not achieved after adjusting for covariates (Supplementary Table 9b).

## Discussion

The pancreatic vascular system is complex, and preoperative imaging assessment is necessary. We used PAAF-PVV to ensure a precise preoperative pancreatic vascular assessment. The performance of the same surgeon with and without the PAAF-PVV tool was compared, which led to inevitable temporal bias. However, the operating surgeon had performed over 100 pancreatic surgeries as the primary operator at the start of the study, having already passed the learning curve. The Pancreatic Surgery Department at PUMCH is one of the earliest established pancreatic surgery centers in China. Its perioperative management strategy for pancreatic patients is highly standardized, with no significant changes in the perioperative management protocol during the study period. Thus, we believe the study results remain interpretable. Here, we found no significant difference in intraoperative blood loss and postoperative PPH. But the exploratory analysis indicator - ΔHb_POD2-POD1 in the group of patients receiving distal pancreatic surgery show significant difference. Although the drop of postoperative hemoglobin levels is not a direct indicator, we believe ΔHb within the first three PODs can partially reflect bleeding attributable to surgery-related factors, particularly the meticulousness of intraoperative vascular dissection and the thoroughness of hemostasis, for the following reasons: (1) The recording of intraoperative blood loss is influenced by the surgeon’s subjective estimation and PPH is also confounded by factors such as pancreatic fistula and infection. Therefore, a more objective indicator is probably needed; (2) Postoperative management for pancreatic surgery patients in our center is highly standardized (see Methods section for details), thereby minimizing the impact of postoperative fluid management on hemoglobin levels; (3) Only two patients received postoperative transfusion therapy prior to 7am on POD2, minimally impacting the ΔHb_POD2-POD1 analysis (Supplementary Table 10); (4) ΔHb POD1-Pre was positively related with postoperative transfusion and length of stay, and PPH was also related with postoperative transfusion, postoperative intervention and length of stay (Tables 4 and 5; Supplementary Table 5a,6). These were attributable to the substantial intraoperative hemorrhage. To rectify the patients’ hypochromic status, clinicians opted for early blood transfusion therapy. Consequently, a statistically significant difference was observed in ΔHb POD1-Pre, whereas no statistical significance could be achieved for ΔHb POD2-POD1 and ΔHb POD3-POD1. However, robust statistical evidence supporting the bleeding-reducing effect of this protocol is lacking in the overall study. Furthermore, PAAF-PVV may only be suitable for specific surgeon groups, such as trainees or newly independent pancreatic surgeons, helping them navigate the learning curve. It might not provide significant added value for highly experienced surgeons. The generalization of this PAAF-PVV application experience is more applicable to high-volume, experienced pancreatic centers. Given the precise perioperative management and experienced surgical team at PUMCH, the application of PAAF-PVV may need to be adjusted based on local clinical practices and surgeon expertise when extended to low-volume centers or different regions. Thus, the value of PAAF-PVV is currently more theoretical than practical.

DPA is one of the primary arteries feeding the body and tail of the pancreas. Previously, DPA was recognized as the inferior or superior pancreatic artery coursing transversely along the caudal margin of the pancreas [11]. Currently, DPA has been studied thoroughly, and its anatomical complexity presents significant challenges for intraoperative management. The reported prevalence of the DPA is 95.8% [5], while our study reports a prevalence of approximately 88%. Numerous studies have revealed the variable origins and branching patterns of the DPA, yet no universally accepted classification system exists for DPA variability. In this study, we proposed a simplified classification system, categorizing DPA origins into five types and further subdividing them into detailed subtypes. While this minimalist classification scheme does not account for potential anastomoses with other arterial branches or variations in course trajectories, it substantially reduces the complexity of DPA typing and offers greater clinical applicability. For instance, some patients may have the DPA arising from an aRHA [12]. Surgeons need to be cautious and preserve the aRHA during surgery to prevent postoperative liver necrosis and liver failure. In this study, 4.2% (7/166) of DPA was identified as arising from aRHA, which we classified as Type III, providing a valuable warning to surgeons. However, the sample sizes for specific DPA origin types were small, particularly for type III and type IV (0.6%, 1/166), which introduces challenges for subsequent clinical analysis and increases the potential for random error. Furthermore, *Sharma S et al.* reported that a single DPA was visualized in most of the patients (*n* = 484, 99.1%) while a double origin of the DPA observed in four cases [13]. In our study, we encountered one patient with a DPA originating from both the SpA and SMA as identified by preoperative ceCT, which was confirmed during surgery. This rare double origin was not included in our classification system.

Previous studies have broadly classified DPA branches into left and right branches, with the left branch supplying the body and tail of the pancreas and the right branch supplying the head and uncinate process. However, different DPA origins result in more complex branching patterns. From a practical perspective, we briefly classified DPA branches into four categories (HB, FB, UB and ExB) without considering its origin. FB is the most common DPA branch with 90.8%. *Masahiro Yamane et al.* reported 86% of the inferior branch (equivalent to FB) of DPA which also accounted for the largest proportion [8]. Existing studies reported a low prevalence of HB (32%), but our findings demonstrated that HB presented in up to 73% of cases. Moreover, previous literature has primarily described ExB as supplying colic branches. However, our study revealed that the ExB could also serve as a branch supplying the small intestine. Thus, we refined the ExB into the CB, JB and IPDB, which effectively guides the management of these variations during surgery.

We noted that type IC DPA was associated with greater intraoperative blood loss, which is difficult to interpret but may be due to its longer and more concealed course. Though DPA is not the most common source of postoperative arterial bleeding, bleeding from DPA is hard to treat due to its anatomical variations in origin and branches which leads to difficulty for super-selection of arteriography [14]. We found that type IIB DPA was associated with more early postoperative hemoglobin decline. Type IIB DPA originates from a branch of the superior mesenteric artery - most commonly the MCA, IPDA, or JA1. These arteries lie within the central dissection zone of pancreatoduodenectomy, a region characterized by a large surgical wound area and a high risk of postoperative arterial bleeding, and therefore requires meticulous and precise surgical handling [15]. This may explain why patients with type IIB DPA exhibited relatively low intraoperative blood loss yet experienced the greatest early postoperative decline in hemoglobin.

Additionally, DPA-HB may also be associated with early postoperative bleeding in group undergoing distal pancreatic surgery. The HB courses on the cranial side of the SpV, and laparoscopic pancreatic surgery usually begins from the caudal side, so HB is exposed later than FB. When dissecting the upper edge of the pancreas, an ultrasonic scalpel is often used to cut directly without carefully dissecting the small blood vessels there. *Alhazemi* et al. previously reported that the bleeding branch of DPA included the trunk, left transverse pancreatic branch that referred to HB and FB in our study, uncinate process branch and anastomotic branch to the right gastroepiploic artery [16]. Thus, careful preoperative evaluation of DPA branches and delicately management are necessary for the prevention of postoperative bleeding from DPA. Moreover, some surgeons currently cut the root of the DPA directly during surgery to reduce the risk of bleeding. *Chong-Yi Jiang et al.* found that the patients of early DPA ligation showed a significantly lower mean blood loss [17]. If the root of the DPA is directly identified and cut, then the influence of different DPA branches on surgical bleeding would no longer exist. Therefore, future studies should further expand the cohort to investigate the effects of ligating the DPA at its root on intraoperative and postoperative bleeding.

The IPDV primarily exhibits three drainage types: the first jejunal venous trunk (JVT1), the superior mesenteric vein (SMV), or a combination of JVT1 and SMV (dual drainage). Our previous research indicated that in patients undergoing pancreatoduodenectomy, IPDV flowing into JVT1 was associated with greater intraoperative blood loss [18]. However, while the current study showed a similar trend, it did not reach statistical significance. The CIPV serves as the main drainage vessel for the pancreatic body and tail, typically emptying into the SMV, splenic vein (SpV), or inferior mesenteric vein (IMV). Our study further observed that the CIPV could drain into the IMV, SMV, SpV, JVT, or colic veins. Rare studies have reported an association between CIPV variant types and surgery-related bleeding. Our results demonstrate that CIPV draining into the IMV was correlated with early postoperative hemoglobin decline. IMV-type CIPV drainage involves venous structures with more complex anatomical trajectories, which are more fragile and technically challenging to dissect during surgery [19]. Given that our data showed a significant ΔHb decline but no corresponding increase in PPH incidence, we propose that this drainage pattern may predispose patients to subclinical bleeding (e.g., slow venous oozing that is not severe enough to meet PPH criteria but sufficient to cause a measurable drop in hemoglobin). These finding warrants attention in clinical practice, as occult bleeding may still impact patient recovery and require closer postoperative monitoring.

In addition, this study utilizes 3D reconstruction technology and 3D printed models to illustrate typical DPA variation types. 3D visualization can be used in pancreatic surgery to achieve preoperative accurately evaluation, individualized surgical plan formulation, surgical teaching, intraoperative positioning and even achieve postoperative complication prediction [20, 21]. However, their current value lies more in education and training. The development of real-time intraoperative intelligent navigation models and virtual-reality fusion technology in the future will offer more substantial support for intraoperative decision-making.

The study has additional limitations. Some analyses involved relatively small sample sizes, which inevitably introduced bias. In fact, we performed multiple comparison corrections or multivariable analysis for all positive findings, yet most of them did not remain statistically significant. This is likely attributable to the limited sample sizes and the presence of multiple confounding factors. Due to the limited sample size, further stratified analysis was not feasible. Nevertheless, comprehensive multivariate analysis with covariate adjustment was performed, which demonstrated no statistically significant differences. The evaluation of DPA is also influenced by the radiological expertise of surgeons. It is also important to acknowledge that several other important factors contribute to bleeding during pancreatic surgery, including other vascular handling, gastrointestinal reconstruction, pancreatic fistula, and infection-related complications. In practice, for more experienced pancreatic surgeons, surgery-related bleeding is more closely associated with vascular management expertise and technique rather than vascular classification itself. However, less experienced young surgeons who often adhere to a fixed operative approach, possess limited vascular handling experience, and have not yet reached peak technical proficiency, may face varying bleeding risks when encountering different vascular variants, as a uniform surgical strategy may not adequately address anatomical variations. Regardless, this study provides valuable clinical insights, which may help enhance surgical safety in pancreatic procedures.

## Supplementary Information

Supplementary Material 1.

Supplementary Material 2.

Supplementary Material 3.

## Data Availability

The datasets used and analyzed during the current study are available from the corresponding author on reasonable request.

## Abbreviations

ANOVA: analysis of variance
AR: augmented reality
CA: celiac trunk/artery
CB: colic artery branch
ceCT: contrast-enhanced computed tomography
CHA: common hepatic artery
CIPV: centro-inferior pancreatic vein
DP: distal pancreatectomy
DPA: dorsal pancreatic artery
DPRHP: duodenum preserving resection of head of pancreas
EN: enucleation
ExB: extra branches
FB: foot-side branch
GDA: gastroduodenal artery
HB: head-side branch
HU: Hounsfield Unit
IMV: inferior mesenteric vein
IOH: intraoperative hemorrhage
IPDA: inferior pancreaticoduodenal artery
IPDB: inferior pancreaticoduodenal artery branch
IPDV: inferior pancreaticoduodenal vein
JA: jejunal artery
JB: jejunal artery branch
JVT: jejunal venous trunk
LCA: left colic artery
LCV: left colic vein
LGA: left gastric artery
MCA: middle colic artery
MCV: middle colic vein
PAAF-PVV: Preoperative Accurate Assessment Form for Pancreatic Vascular Variations
P/A-SPDA: posterior/anterior superior pancreaticoduodenal artery
PD: pancreaticoduodenectomy
PPH: post-pancreatectomy hemorrhage
PUMCH: Peking Union Medical College Hospital
RCA: right colic artery
RCV: right colic vein
rRHA: replaced right hepatic artery
S/I-PDA: superior/inferior pancreaticoduodenal arteries
SMA: superior mesenteric artery
SMV: superior mesenteric vein
SpA: splenic artery
SpV: splenic vein
TP: total pancreatectomy
UB: uncinate process branch
VR: virtual reality
