# Supplementary material for: Impact of precise preoperative vascular assessment and different dorsal pancreatic artery variant subtypes on pancreatic surgery-related bleeding
Source: BMC Gastroenterol. 2026 Feb 14;26:186. doi: 10.1186/s12876-026-04687-8 (PMC13011292; doi:10.1186/s12876-026-04687-8)
Supplement: Supplementary file 1 — Supplementary Material 1. [file 12876_2026_4687_MOESM1_ESM.pdf]

PAAF-PVV for PD

Name: ID: Diagnosis: Date:			▯ Preoperative assessment by ceCT;<br>▮ Intraoperative assessment directly;<br>[ ] Numbers of branches;<br>[A] A repersents "did not expose";<br>[B] B represents "Indeterminate";
Surgery Method	Preoperative	a. PD b. DPRHP c. Others_______<br>a. open b. Laparoscope c. Robotic	
	Intraoperative	a. PD b. DPRHP c.Others_______<br>a. open b. Laparoscope c. Robotic	Surgical approach:

Vessels		Assessment	Match Degree
CA	Stenosis	□ Yes □ No □ Other_________<br>□ Yes □ No □ Other_________	a. Fully<br>b. Potential<br>c. Mismatched<br>d. Non-assessable
CHA	Origin	□ CA □ SMA □ Other_________<br>□ CA □ SMA □ Other_________	a. Fully<br>b. Potential<br>c. Mismatched<br>d. Non-assessable
aRHA	Origin [Number]	□ None □ GDA [ ] □ SMA [ ] □ Other_________ [ ]<br>□ None □ GDA [ ] □ SMA [ ] □ Other_________ [ ]	a. Fully<br>b. Potential<br>c. Mismatched<br>d. Non-assessable
aLHA	Origin [Number]	□ None □ LGA [ ] □ SMA [ ] □ Other_________ [ ]<br>□ None □ LGA [ ] □ SMA [ ] □ Other_________ [ ]	a. Fully<br>b. Potential<br>c. Mismatched<br>d. Non-assessable
GDA	Origin [Number]	□ CHA [ ] □ CA [ ] □ SMA [ ] □ Other_________ [ ]<br>□ CHA [ ] □ CA [ ] □ SMA [ ] □ Other_________ [ ]	a. Fully<br>b. Potential<br>c. Mismatched<br>d. Non-assessable
A-SPDA	Origin [Number]	□ GDA [ ] □ CHA [ ] □ Other_________ [ ] □ None<br>□ GDA [ ] □ CHA [ ] □ Other_________ [ ] □ None	a. Fully<br>b. Potential<br>c. Mismatched<br>d. Non-assessable
P-SPDA	Origin [Number]	□ GDA [ ] □ CHA [ ] □ Other_________ [ ] □ None<br>□ GDA [ ] □ CHA [ ] □ Other_________ [ ] □ None	a. Fully<br>b. Potential<br>c. Mismatched<br>d. Non-assessable
IPDA	Origin [Number]	□ SMA [ ] □ JA1 [ ] □ JA2 [ ] □ JA3 [ ] □ DPA [ ]<br>□ RHA [ ] □ RHA [ ] □ Other________ [ ] □ None<br>□ SMA [ ] □ JA1 [ ] □ JA2 [ ] □ JA3 [ ] □ DPA [ ]<br>□ RHA [ ] □ aRHA [ ] □ Other________ [ ] □ None	a. Fully<br>b. Potential<br>c. Mismatched<br>d. Non-assessable
A-IPDA	Origin [Number]	□ SMA [ ] □ JA1 [ ] □ JA2 [ ] □ JA3 [ ] □ DPA [ ]<br>□ RHA [ ] □ Other________ [ ] □ None<br>□ SMA [ ] □ JA1 [ ] □ JA2 [ ] □ JA3 [ ] □ DPA [ ]<br>□ RHA [ ] □ Other________ [ ] □ None	a. Fully<br>b. Potential<br>c. Mismatched<br>d. Non-assessable
P-IPDA	Origin [Number]	□ SMA [ ] □ JA1 [ ] □ JA2 [ ] □ JA3 [ ] □ DPA [ ]<br>□ RHA [ ] □ Other________ [ ] □ None<br>□ SMA [ ] □ JA1 [ ] □ JA2 [ ] □ JA3 [ ] □ DPA [ ]<br>□ RHA [ ] □ Other________ [ ] □ None	a. Fully<br>b. Potential<br>c. Mismatched<br>d. Non-assessable

DPA	Origin [Number]	□ SpA [ ] □ CHA-CA [ ] □ SMA [ ] □ GDA [ ] □ aRHA [ ]<br>□ JA1 [ ] □ IPDA [ ] □ RGA [ ] □ RGEA [ ] □ Other____ [ ] □ None<br>□ SpA [ ] □ CHA-CA [ ] □ SMA [ ] □ GDA [ ] □ aRHA [ ]<br>□ JA1 [ ] □ IPDA [ ] □ RGA [ ] □ RGEA [ ] □ Other____ [ ] □ None	a. Fully<br>b. Potential<br>c. Mismatched<br>d. Non-assessable
	Branches [Number]	□ HB [ ] □ FB [ ] □ UB [ ] □ IPDA [ ] □ Other_________ [ ]<br>□ HB [ ] □ FB [ ] □ UB [ ] □ IPDA [ ] □ Other_________ [ ]	a. Fully<br>b. Potential<br>c. Mismatched<br>d. Non-assessable
RGA	Origin [Number]	□ CHA [ ] □ PHA [ ] □ LHA [ ] □ RHA [ ] □ GDA [ ] □ Other_______<br>□ CHA [ ] □ PHA [ ] □ LHA [ ] □ RHA [ ] □ GDA [ ] □ Other_______	a. Fully<br>b. Potential<br>c. Mismatched<br>d. Non-assessable
	Shared trunk with [Number]	□ None □ SDA [ ] □ Other______ [ ]<br>□ None □ SDA [ ] □ Other______ [ ]	a. Fully<br>b. Potential<br>c. Mismatched<br>d. Non-assessable
Henle干	Inflow	□ None □ SMV [ ] □ Other_______<br>□ None □ SMV [ ] □ Other_______	a. Fully<br>b. Potential<br>c. Mismatched<br>d. Non-assessable
	Branches [Number]	□ REGV [ ] □ SRCV [ ] □ ASPDV [ ] □ AIPDV [ ] □ MCV [ ] □ Other_______<br>□ REGV [ ] □ SRCV [ ] □ ASPDV [ ] □ AIPDV [ ] □ MCV [ ] □ Other_______	a. Fully<br>b. Potential<br>c. Mismatched<br>d. Non-assessable

A-SPDV	Inflow [Number]	□ Henle's T [ ] □ RGEV [ ] □ SMV [ ] □ Other________ [ ] □ None	a. Fully<br>b. Potential<br>c. Mismatched<br>d. Non-assessable
		□ Henle's T [ ] □ RGEV [ ] □ SMV [ ] □ Other________ [ ] □ None	
P-SPDV	Inflow [Number]	□ PV [ ] □ RGV [ ] □ Other________ [ ] □ None	a. Fully<br>b. Potential<br>c. Mismatched<br>d. Non-assessable
		□ PV [ ] □ RGV [ ] □ Other________ [ ] □ None	
FJT	Position relative to SMA	□ Dorsal □ Ventral □ Other________ □ None	a. Fully<br>b. Potential<br>c. Mismatched<br>d. Non-assessable
		□ Dorsal □ Ventral □ Other________ □ None	
SJT	Position relative to SMA	□ Dorsal □ Ventral □ Other________ □ None	a. Fully<br>b. Potential<br>c. Mismatched<br>d. Non-assessable
		□ Dorsal □ Ventral □ Other________ □ None	
TJT	Position relative to SMA	□ Dorsal □ Ventral □ Other________ □ None	a. Fully<br>b. Potential<br>c. Mismatched<br>d. Non-assessable
		□ Dorsal □ Ventral □ Other________ □ None	
IPDV	Inflow [Number]	□ SMV [ ] □ FJT [ ] □ SJT [ ] □ TJT [ ] □ Other___ [ ] □ None	a. Fully<br>b. Potential<br>c. Mismatched<br>d. Non-assessable
		□ SMV [ ] □ FJT [ ] □ SJT [ ] □ TJT [ ] □ Other___ [ ] □ None	
A-IPDV	Inflow [Number]	□ SMV [ ] □ FJT [ ] □ SJT [ ] □ TJT [ ] □ Other___ [ ] □ None	a. Fully<br>b. Potential<br>c. Mismatched<br>d. Non-assessable
		□ SMV [ ] □ FJT [ ] □ SJT [ ] □ TJT [ ] □ Other___ [ ] □ None	
P-IPDV	Inflow [Number]	□ SMV [ ] □ FJT [ ] □ SJT [ ] □ TJT [ ] □ Other___ [ ] □ None	a. Fully<br>b. Potential<br>c. Mismatched<br>d. Non-assessable
		□ SMV [ ] □ FJT [ ] □ SJT [ ] □ TJT [ ] □ Other___ [ ] □ None	
LGV	Inflow [Number]	□ PV [ ] □ SpV [ ] □ Bifurc [ ] □ IMV [ ] □ Other_____ [ ]	a. Fully<br>b. Potential<br>c. Mismatched<br>d. Non-assessable
		□ PV [ ] □ SpV [ ] □ Bifurc [ ] □ IMV [ ] □ Other_____ [ ]	
	Course	□ Dorsal CHA course □ Ventral CHA course □ Ventral SpA course □ Dorsal SpA course □ Other_____	a. Fully<br>b. Potential<br>c. Mismatched<br>d. Non-assessable
		□ Dorsal CHA course □ Ventral CHA course □ Ventral SpA course □ Dorsal SpA course □ Other_____	

RGV	Inflow [Number]	□ PV [ ] □ SpV [ ] □ IMV [ ] □ Other_____ [ ]	a. Fully<br>b. Potential<br>c. Mismatched<br>d. Non-assessable
		□ PV [ ] □ SpV [ ] □ IMV [ ] □ Other_____ [ ]	
aberrant RGV	Inflow [Number]	□ None □ PV Trunk [ ] □ Left branch of PV [ ] □ directly to liver [ ] □ Other___________	a. Fully<br>b. Potential<br>c. Mismatched<br>d. Non-assessable
		□ None □ PV Trunk [ ] □ Left branch of PV [ ] □ directly to liver [ ] □ Other___________	
CIPV	Inflow [Number]	□ SMV [ ] □ SpV [ ] □ IMV [ ] □ CMV [ ] □ Other____________	a. Fully<br>b. Potential<br>c. Mismatched<br>d. Non-assessable
		□ SMV [ ] □ SpV [ ] □ IMV [ ] □ CMV [ ] □ Other____________	
IMV	Inflow [Number]	□ SMV [ ] □ SpV [ ] □ Bifurc [ ] □ Other _______ [ ]	a. Fully<br>b. Potential<br>c. Mismatched<br>d. Non-assessable
		□ SMV [ ] □ SpV [ ] □ Bifurc [ ] □ Other _______ [ ]	
	Shared trunk with [Number]	□ LCV [ ] □ RCV [ ] □ MCV [ ] □ Other _______ [ ]	a. Fully<br>b. Potential<br>c. Mismatched<br>d. Non-assessable
		□ LCV [ ] □ RCV [ ] □ MCV [ ] □ Other _______ [ ]	
SpV	Course	□ Fully extrapancreatic □ Partially pancreas-covered □ Superficially pancreas-embedded □ Other ________	a. Fully<br>b. Potential<br>c. Mismatched<br>d. Non-assessable
		□ Fully extrapancreatic □ Partially pancreas-covered □ Superficially pancreas-embedded □ Other ________	
	Block	□ None occlusion □ Partially occlusion □ Fully occlusion<br>(a.Compression; b.Tumor invasion; c.Thrombus; d.Tumor thrombus; e.Spasm; f.Other___)	a. Fully<br>b. Potential<br>c. Mismatched<br>d. Non-assessable
		□ None occlusion □ Partially occlusion □ Fully occlusion<br>(a.Compression; b.Tumor invasion; c.Thrombus; d.Tumor thrombus; e.Spasm; f.Other___)	
PPH	□ Yes □ No	□ SGV→Gastric body/fundus→Azygos V □ SGV→Gastric body/fundus→Coronary V→PV<br>□ SGV→Phrenic V→Intercostal V→IVC □ LGEV-RGEV→SMV/PV □ LGEV-RGEV→Colonic V→IMV □ Other____	a. Fully<br>b. Potential<br>c. Mismatched<br>d. Non-assessable
	□ Yes □ No	□ SGV→Gastric body/fundus→Azygos V □ SGV→Gastric body/fundus→Coronary V→PV<br>□ SGV→Phrenic V→Intercostal V→IVC □ LGEV-RGEV→SMV/PV □ LGEV-RGEV→Colonic V→IMV □ Other____	

Note:
① Indeterminate represents: "CT-indeterminate: Uncertain on CT evaluation" or "Intraop-indeterminate: Identifiable during surgery but uncertain" or "CT-guided identification: Not initially found intraoperatively but eventually located with CT guidance".
② Matched: Preoperative CT assessment "fully visible" = Intraoperative assessment "fully visible", complete concordance between imaging and surgical findings.
③ Potentially Matched: Preoperative CT "visible/indeterminate" ≈ Intraoperative "visible/indeterminate", qualitative agreement without absolute certainty.
④ Mismatched: Preoperative CT "visible/indeterminate" ≠ Intraoperative "visible/indeterminate", discordant findings between imaging and surgery.
⑤ Non-assessable: Due to poor vascular visualization or intraoperative non-visualization and technical limitations in exposure, precludes reliable matching evaluation.
⑥ PPH: Pancreatic portal hypertension
Discussion:

PAAF-PVV for DP

Name: ID: Diagnosis: Date:			▯ Preoperative assessment by ceCT;<br>▮ Intraoperative assessment directly;<br>[ ] Numbers of branches;<br>[A] A repersents "did not expose";<br>[B] B represents "did not expose well";
Surgery Method	Preoperative	a. DPS d. Kimura e. Warshaw	
		a. open b. Laparoscope c. Robotic	
	Intraoperative	a. DPS d. Kimura e. Warshaw	
		a. open b. Laparoscope c. Robotic	Surgical approach:

Vessels		Assessment	Match Degree
CA	Stenosis	□ Yes □ No □ Other________<br>□ Yes □ No □ Other________	a. Fully<br>b. Potential<br>c. Mismatched<br>d. Non-assessable
CHA	Origin	□ CA □ SMA □ Other_________<br>□ CA □ SMA □ Other_________	a. Fully<br>b. Potential<br>c. Mismatched<br>d. Non-assessable
aRHA	Origin [Number]	□ None □ GDA [ ] □ SMA [ ] □ Other_________ [ ]<br>□ None □ GDA [ ] □ SMA [ ] □ Other_________ [ ]	a. Fully<br>b. Potential<br>c. Mismatched<br>d. Non-assessable
aLHA	Origin [Number]	□ None □ LGA [ ] □ SMA [ ] □ Other_________ [ ]<br>□ None □ LGA [ ] □ SMA [ ] □ Other_________ [ ]	a. Fully<br>b. Potential<br>c. Mismatched<br>d. Non-assessable
DPA	Origin [Number]	□ SpA [ ] □ CHA-CA [ ] □ SMA [ ] □ GDA [ ] □ aRHA [ ]<br>□ JA1 [ ] □ IPDA [ ] □ RGA [ ] □ RGEA [ ] □ Other____ [ ] □ None<br>□ SpA [ ] □ CHA-CA [ ] □ SMA [ ] □ GDA [ ] □ aRHA [ ]<br>□ JA1 [ ] □ IPDA [ ] □ RGA [ ] □ RGEA [ ] □ Other____ [ ] □ None	a. Fully<br>b. Potential<br>c. Mismatched<br>d. Non-assessable
	Branch [Number]	□ HB [ ] □ FB [ ] □ UB [ ] □ IPDA [ ] □ Other_________ [ ]<br>□ HB [ ] □ FB [ ] □ UB [ ] □ IPDA [ ] □ Other_________ [ ]	a. Fully<br>b. Potential<br>c. Mismatched<br>d. Non-assessable
SpA	Course	□ Fully extrapancreatic □ Partially pancreas-covered □ Superficially pancreas-embedded □ Other ________<br>□ Fully extrapancreatic □ Partially pancreas-covered □ Superficially pancreas-embedded □ Other ________	a. Fully<br>b. Potential<br>c. Mismatched<br>d. Non-assessable
	Branch along the way [Number]	□ None [ ] □ 1 [ ] □ 2 [ ] □ 3 [ ] □ 4 [ ] □ Other [ ]<br>□ None [ ] □ 1 [ ] □ 2 [ ] □ 3 [ ] □ 4 [ ] □ Other [ ]	a. Fully<br>b. Potential<br>c. Mismatched<br>d. Non-assessable
	Terminal bifurcation	□ Prepancreatic tail [ ] □ Pancreaticosplenic junction [ ] □ Splenic hilum [ ]<br>□ Prepancreatic tail [ ] □ Pancreaticosplenic junction [ ] □ Splenic hilum [ ]	a. Fully<br>b. Potential<br>c. Mismatched<br>d. Non-assessable
LGEA	Course in Spleen hilum	□ Fully extrapancreatic □ Partially pancreas-covered □ Superficially pancreas-embedded □ Other ________<br>□ Fully extrapancreatic □ Partially pancreas-covered □ Superficially pancreas-embedded □ Other ________	a. Fully<br>b. Potential<br>c. Mismatched<br>d. Non-assessable

Henle干	Inflow	□ None □ SMV [ ] □ Other_______<br>□ None □ SMV [ ] □ Other_______	a. Fully<br>b. Potential<br>c. Mismatched<br>d. Non-assessable
	Branches [Number]	□ REGV [ ] □ SRCV [ ] □ ASPDV [ ] □ AIPDV [ ] □ MCV [ ] □ Other_______<br>□ REGV [ ] □ SRCV [ ] □ ASPDV [ ] □ AIPDV [ ] □ MCV [ ] □ Other_______	a. Fully<br>b. Potential<br>c. Mismatched<br>d. Non-assessable
LGV	Inflow [Number]	□ PV [ ] □ SpV [ ] □ Bifurc [ ] □ Other________ [ ]<br>□ PV [ ] □ SpV [ ] □ Bifurc [ ] □ Other________ [ ]	a. Fully<br>b. Potential<br>c. Mismatched<br>d. Non-assessable
	Course	□ Dorsal CHA course □ Ventral CHA course □ Ventral SpA course □ Dorsal SpA course □ Other______<br>□ Dorsal CHA course □ Ventral CHA course □ Ventral SpA course □ Dorsal SpA course □ Other______	a. Fully<br>b. Potential<br>c. Mismatched<br>d. Non-assessable
CIPV	Inflow [Number]	□ SMV [ ] □ SpV [ ] □ IMV [ ] □ CMV [ ] □ Other____________<br>□ SMV [ ] □ SpV [ ] □ IMV [ ] □ CMV [ ] □ Other____________	a. Fully<br>b. Potential<br>c. Mismatched<br>d. Non-assessable
IMV	Inflow [Number]	□ SMV [ ] □ SpV [ ] □ Bifurc [ ] □ Other _________ [ ]	a. Fully<br>b. Potential

IMV	Inflow [Number]	□ SMV [ ] □ SpV [ ] □ Bifurc [ ] □ Other _________ [ ]	c. Mismatched<br>d. Non-assessable
SpV	Course	□ Fully extrapancreatic □ Partially pancreas-covered □ Superficially pancreas-embedded □ Other ________<br>□ Fully extrapancreatic □ Partially pancreas-covered □ Superficially pancreas-embedded □ Other ________	a. Fully<br>b. Potential<br>c. Mismatched<br>d. Non-assessable
	Occlusion	□ None occlusion □ Partially occlusion □ Fully occlusion<br>(a.Compression; b.Tumor invasion; c.Thrombus; d.Tumor thrombus; e.Spasm; f.Other___)<br>□ None occlusion □ Partially occlusion □ Fully occlusion<br>(a.Compression; b.Tumor invasion; c.Thrombus; d.Tumor thrombus; e.Spasm; f.Other___)	a. Fully<br>b. Potential<br>c. Mismatched<br>d. Non-assessable
LGEV	Course	□ Fully extrapancreatic □ Partially pancreas-covered □ Superficially pancreas-embedded □ Other ________<br>□ Fully extrapancreatic □ Partially pancreas-covered □ Superficially pancreas-embedded □ Other ________	a. Fully<br>b. Potential<br>c. Mismatched<br>d. Non-assessable
PPH	□ Yes □ No	□ SGV→Gastric body/fundus→Azygos V □ SGV→Gastric body/fundus→Coronary V→PV<br>□ SGV→Phrenic V→Intercostal V→IVC □ LGEV-RGEV→SMV/PV □ LGEV-RGEV→Colonic V→IMV □ Other____	a. Fully<br>b. Potential<br>c. Mismatched<br>d. Non-assessable
	□ Yes □ No	□ SGV→Gastric body/fundus→Azygos V □ SGV→Gastric body/fundus→Coronary V→PV<br>□ SGV→Phrenic V→Intercostal V→IVC □ LGEV-RGEV→SMV/PV □ LGEV-RGEV→Colonic V→IMV □ Other____	a. Fully<br>b. Potential<br>c. Mismatched<br>d. Non-assessable

Note:
① Indeterminate represents: "CT-indeterminate: Uncertain on CT evaluation" or "Intraop-indeterminate: Identifiable during surgery but uncertain" or "CT-guided identification: Not initially found intraoperatively but eventually located with CT guidance".
② Matched: Preoperative CT assessment "fully visible" = Intraoperative assessment "fully visible", complete concordance between imaging and surgical findings.
③ Potentially Matched: Preoperative CT "visible/indeterminate" ≈ Intraoperative "visible/indeterminate", qualitative agreement without absolute certainty.
④ Mismatched: Preoperative CT "visible/indeterminate" ≠ Intraoperative "visible/indeterminate", discordant findings between imaging and surgery.
⑤ Non-assessable: Due to poor vascular visualization or intraoperative non-visualization and technical limitations in exposure, precludes reliable matching evaluation.
⑥ PPH: Pancreatic portal hypertension
Discussion:
