# Supplementary material for: Impact of precise preoperative vascular assessment and different dorsal pancreatic artery variant subtypes on pancreatic surgery-related bleeding
Source: BMC Gastroenterol. 2026 Feb 14;26:186. doi: 10.1186/s12876-026-04687-8 (PMC13011292; doi:10.1186/s12876-026-04687-8)
Supplement: Supplementary file 2 — Supplementary Material 2. [file 12876_2026_4687_MOESM2_ESM.docx]

**Supplementary Table 1: The DPA origin matching degree based on preoperative assessment and intraoperative findings.**

| Matching grade | N | Ratio | Note |
| --- | --- | --- | --- |
| Fully matched | 44 | 74.6% | Preoperative assessment result was the same as that in intraoperative assessment. |
| Potentially matched | 21 | 35.6% | Preoperative assessment result was the same as intraoperative assessment, but uncertain, which was related to inadequate intraoperative exposure or unnecessary intraoperative exposure. |
| Mismatched | 3 | 5.1% | Case1: DPA was preoperatively found to possibly originate from left colonic artery (LCA) , but intraoperatively not found. Further, postoperative re-evaluation of preoperative CT images confirmed the absence of DPA and the preoperative misjudgment.  Case2: DPA was preoperatively not found, but intraoperatively found to originate from SpA. Further, postoperative re-evaluation of the preoperative CT imaging revealed a DPA originating from the root of the SpA and coursing posteriorly and inferiorly on coronal reconstruction.  Case3: DPA was preoperatively found to originate from SpA, but intraoperatively found to originate from aRHA.Further, postoperative re-evaluation of preoperative CT imaging revealed that the DPA originated from an aberrant RHA and bifurcated into two branches. |

**Supplementary Table 2: The DPA origin types based on intraoperative findings.**

| Types | N | Ratio |
| --- | --- | --- |
| I | 29 | 42.6% |
| IA | 16 | 23.5% |
| IB | 11 | 16.2% |
| IC | 2 | 2.9% |
| II | 23 | 33.8% |
| IIA | 17 | 25.0% |
| IIB | 6 | 8.8% |
| III | 5 | 7.4% |
| IV | 0 | 0.0% |
| V | 11 | 16.2% |

**Supplementary Table 3: The DPA branches matching degree based on preoperative assessment and intraoperative findings.**

| Matching grade | N | Ratio |
| --- | --- | --- |
| Fully matched | 47 | 46.1% |
| Potentially matched | 48 | 47.1% |
| Mismatched | 7 | 6.9% |

**Supplementary Table 4: The DPA branches distribution based on intraoperative findings**

| Types | N | Ratio |
| --- | --- | --- |
| HB | 83 | 81.4% |
| FB | 93 | 91.2% |
| UB | 79 | 77.5% |
| ExB | 13 | 12.7% |
| IPDB | 8 | 7.8% |
| JB | 1 | 1.0% |
| CB | 4 | 3.9% |

**Supplementary Table 5a: The analysis of PPH and intraoperative bleeding-related factors in patients undergoing proximal pancreatic surgery**

| DPA variation | | PPH | | | Intraoperative bleeding (mL) | | | ΔHb (POD1-Pre) (g/L) | | |
| --- | --- | --- | --- | --- | --- | --- | --- | --- | --- | --- |
|  |  | No | Yes | P | N | Mean±SD | P | N | Mean±SD | P |
| Gender | Female | 22 (46.8%) | 3 (25.0%) | 0.172 | 25 | 477.60±454.728 | 0.828 | 25 | -8.32±15.041 | 0.083 |
|  | Male | 25 (53.2%) | 9 (75.0%) |  | 34 | 506.47±534.766 |  | 34 | -15.47±15.658 |  |
| Age | years | 61.47±10.705 | 62.42±14.100 | 0.799 | R=0.061,P=0.648 | | | R=0.112,P=0.400 | | |
| Surgical method | Open | 8 (17.0%) | 2 (16.7%) | 0.281 | 10 | 1040±661.984 | **＜0.001** | 10 | -21.60±21.711 | 0.06 |
|  | LAP | 4 (8.5%) | 3 (25.0%) |  | 7 | 492.86±294.998 |  | 7 | -17.14±11.725 |  |
|  | Robotic | 35 (74.5%) | 7 (58.3%) |  | 42 | 364.52±390.760 |  | 42 | -9.48±13.816 |  |
| Pathology type | Benign | 9 (19.1%) | 2 (16.7%) | 0.844 | 11 | 570.91±507.749 | 0.576 | 11 | -21.45±15.699 | **0.033** |
|  | Malignant | 38 (80.9%) | 10 (83.3%) |  | 48 | 476.67±500.091 |  | 48 | -10.37±15.084 |  |
| Origin type | IA | 9 (19.1%) | 4 (33.3%) | 0.680 | 13 | 596.15±551.687 | **0.034^a^** | 13 | -17.00±19.378 | 0.071 |
|  | IB | 6 (12.8%) | 1 (8.3%) |  | 7 | 137.14±100.119 |  | 7 | -18.77±13.198 |  |
|  | **IC** | 4 (8.5%) | 2 (16.7%) |  | **6** | **763.33±927.074** |  | 6 | -0.57±18.082 |  |
|  | IIA | 13 (27.7%) | 2 (16.7%) |  | 15 | 478.67±293.571 |  | 15 | -17.50±19.624 |  |
|  | IIB | 6 (12.8%) | 0 (0%) |  | 6 | 198.33±198.335 |  | 6 | -11.47±12.106 |  |
|  | III | 2 (4.3%) | 1 (8.3%) |  | 3 | 633.33±321.455 |  | 3 | -0.67±6.831 |  |
|  | V | 7 (14.9%) | 2 (16.7%) |  | 9 | 622.22±569.600 |  | 9 | -17.33±10.599 |  |
| Branch number | No | 2 (5.0%) | 0 (0%) | 0.842 | 2 | 725.00±813.173 | 0.56 | 2 | -20.00±0.00 | 0.542 |
|  | 1 | 3 (7.5%) | 0 (0%) |  | 3 | 376.67±304.357 |  | 3 | -5.67±11.015 |  |
|  | 2 | 7 (17.5%) | 2 (20.0%) |  | 9 | 240.00±191.507 |  | 9 | -6.56±10.525 |  |
|  | 3 | 25 (62.5%) | 7 (70.0%) |  | 32 | 522.50±547.793 |  | 32 | -12.12±14.962 |  |
|  | ≥4 | 3 (7.5%) | 1 (10.0%) |  | 4 | 525.00±394.757 |  | 4 | -19.25±27.354 |  |
| FB | No | 5 (12.5%) | 1 (10.0%) | 0.828 | 6 | 413.33±462.241 | 0.76 | 6 | -8.33±11.396 | 0.573 |
|  | Yes | 35 (87.5%) | 9 (90.0%) |  | 44 | 479.09±495.354 |  | 44 | -12.07±15.484 |  |
| HB | No | 10 (25.0%) | 1 (10.0%) | 0.306 | 11 | 537.27±746.928 | 0.616 | 11 | -12.18±11.617 | 0.890 |
|  | Yes | 30 (75.0%) | 9 (90.0%) |  | 39 | 452.56±397.417 |  | 39 | -11.46±15.959 |  |
| UB | No | 9 (22.5%) | 2 (20.0%) | 0.864 | 11 | 528.18±609.554 | 0.665 | 11 | -14.91±14.342 | 0.416 |
|  | Yes | 31 (77.5%) | 8 (80.0%) |  | 39 | 455.13±455.405 |  | 39 | -10.69±15.237 |  |
| ExB | No | 34 (85.0%) | 7 (70.0%) | 0.269 | 41 | 403.17±323.206 | 0.245 | 41 | -9.61±12.673 | 0.166 |
|  | Yes | 6 (15.0%) | 3(30.0%) |  | 9 | 781.11±895.104 |  | 9 | -20.78±21.470 |  |
| IPDV drainage | Type I | 28 (65.1%) | 8(72.7%) | 6330. | 36 | 504.17±491.030 | 0.070 | 36 | -12.89±16.025 | 0.680 |
|  | Non-Type I | 15 (34.9%) | 3(27.3%) |  | 18 | 320.00±241.149 |  | 18 | -11.00±15.239 |  |
| CIPV drainage | IMV | 22 (56.4%) | 4(44.4%) | 0.311 | 26 | 484.23±469.520 | 0.941 | 26 | -11.27±14.529 | 0.722 |
|  | SMV | 6 (15.4%) | 0(0%) |  | 6 | 350.00±250.998 |  | 6 | -11.33±17.761 |  |
|  | SpV | 2 (5.1%) | 0(0%) |  | 2 | 500.00±0.00 |  | 2 | -8.50±6.364 |  |
|  | Colonic vein or JVT | 6 (15.4%) | 3(33.3%) |  | 9 | 392.22±329.081 |  | 9 | -18.78±11.421 |  |
|  | Dual drainage pattern | 3 (7.7%) | 2(22.2%) |  | 5 | 526.00±677.776 |  | 5 | -11.00±17.903 |  |
| CIPV drainage | IMV | 23 (59.0%) | 5(55.6%) | 0.851 | 28 | 467.50±457.777 | 0.818 | 28 | -10.64±14.708 | 0.282 |
|  | Non-IMV | 16 (41.0%) | 4(44.4%) |  | 20 | 438.00±398.347 |  | 20 | -15.20±13.733 |  |
| Postoperative fistula | No | 17 (36.2%) | 0(0%) | **0.003** | 17 | 418.24±359.744 | 0.637 | 17 | -9.88±16.789 | 0.686 |
|  | Biochemical | 18 (38.3%) | 3(25.0%) |  | 21 | 478.10±466.107 |  | 21 | -14.38±13.036 |  |
|  | B/C | 12 (25.5%) | 7(75.0%) |  | 21 | 571.90±623.126 |  | 21 | -12.57±17.546 |  |
| Postoperative infection | No | 39 (83.0%) | 7(58.3%) | 0.066 | 46 | 467.39±402.577 | 0.441 | 46 | -12.24±14.738 | 0.855 |
|  | Yes | 8 (17.0%) | 5(41.7%) |  | 13 | 589.23±762.031 |  | 13 | -13.15±19.308 |  |
| Postoperative transfusion | No | 47 (100%) | 7(58.3%) | **＜0.001** | 54 | 410.37±366.266 | 0.057 | 54 | -10.74±15.107 | **0.005** |
|  | Yes | 0 (0%) | 5(41.7%) |  | 5 | 1400.00±839.643 |  | 5 | -30.80±8.927 |  |
| Postoperative intervention | No | 47 (100%) | 6(50.0%) | **＜0.001** | 53 | 469.81±429.304 | 0.566 | 53 | -12.09±15.218 | 0.618 |
|  | Yes | 0 (0%) | 6(50.0%) |  | 6 | 710.00±950.495 |  | 6 | -15.50±20.744 |  |
| Length of stay | days | 16.47±6.416 | 30.08±20.948 | **0.047** | R=0.126,P=0.340 | | | P=-0.264,**P=0.044** | | |

Note：a. When the variance is uneven, a non-parametric test (Kruskal-Wallis test) is used.

**Supplementary Table 5b: The multiple pairwise comparison analysis of origin type in intraoperative blood loss volume in patients undergoing proximal pancreatic surgery**

|  | Test Statistic | Std. Error | Std. Test Statistic | Sig. P | Adj. Sig. P^a^ |
| --- | --- | --- | --- | --- | --- |
| IB - IIB | -4.333 | 9.494 | -.456 | .648 | 1.000 |
| **IB - IC** | **-20.083** | **9.494** | **-2.115** | **.034** | .722 |
| IB - IIA | -20.700 | 7.811 | -2.650 | .008 | .169 |
| IB - IA | 20.962 | 8.000 | 2.620 | .009 | .185 |
| IB - V | 21.056 | 8.599 | 2.448 | .014 | .301 |
| IB - III | -28.000 | 11.775 | -2.378 | .017 | .366 |
| IIB - IC | 15.750 | 9.852 | 1.599 | .110 | 1.000 |
| IIB - IIA | 16.367 | 8.243 | 1.986 | .047 | .989 |
| IIB - IA | 16.628 | 8.422 | 1.974 | .048 | 1.000 |
| IIB - V | 16.722 | 8.994 | 1.859 | .063 | 1.000 |
| IIB - III | -23.667 | 12.066 | -1.961 | .050 | 1.000 |
| IC - IIA | -.617 | 8.243 | -.075 | .940 | 1.000 |
| IC - IA | .878 | 8.422 | .104 | .917 | 1.000 |
| IC - V | .972 | 8.994 | .108 | .914 | 1.000 |
| IC - III | -7.917 | 12.066 | -.656 | .512 | 1.000 |
| IIA - IA | .262 | 6.466 | .040 | .968 | 1.000 |
| IIA - V | .356 | 7.195 | .049 | .961 | 1.000 |
| IIA - III | -7.300 | 10.792 | -.676 | .499 | 1.000 |
| IA - V | .094 | 7.399 | .013 | .990 | 1.000 |
| IA - III | -7.038 | 10.930 | -.644 | .520 | 1.000 |
| V - III | -6.944 | 11.376 | -.610 | .542 | 1.000 |

a. Significance values have been adjusted by the Bonferroni correction for multiple tests.

**Supplementary Table 6: The analysis of postoperative hemoglobin decline-related factors in patients undergoing proximal pancreatic surgery**

| DPA variation | | ΔHb (POD2-POD1) (g/L) | | | ΔHb (POD3-POD1) (g/L) | | |
| --- | --- | --- | --- | --- | --- | --- | --- |
|  |  | N | Mean±SD | P | N | Mean±SD | P |
| Gender | Female | 22 | -0.86±9.578 | **0.02** | 23 | -5.00±12.049 | **0.034** |
|  | Male | 30 | -8.80±13.134 |  | 29 | -13.52±15.299 |  |
| Age | years | R=0.210,P=0.135 | | | R=0.215,P=0.125 | | |
| Surgical method | Open | 8 | -1.62±6.346 | 0.326 | 10 | -9.00±11.392 | 0.414 |
|  | LAP | 7 | -1.14±6.768 |  | 7 | -16.57±24.959 |  |
|  | Robotic | 37 | -7.08±13.711 |  | 35 | -8.60±12.596 |  |
| Pathology type | Benign | 9 | -1.22±8.105 | 0.262 | 11 | -5.45±9.802 | 0.272 |
|  | Malignant | 43 | -6.33±12.919 |  | 41 | -10.9±15.378 |  |
| Origin type | IA | 12 | -7.50±18.058 | 0.086 | 10 | -8.70±15.931 | 0.501^a^ |
|  | IB | 7 | -8.14±9.477 |  | 6 | -12.17±10.128 |  |
|  | **IC** | **6** | **-0.83±3.430** |  | **6** | **-10.50±13.982** |  |
|  | **IIA** | **12** | **0.67±8.616** |  | **13** | **-3.46±10.564** |  |
|  | **IIB** | **4** | **-20.75±14.221** |  | **5** | **-13.00±5.874** |  |
|  | III | 3 | -6.67±2.082 |  | 3 | -30.67±35.501 |  |
|  | V | 8 | -4.50±8.332 |  | 9 | -9.11±11.028 |  |
| Branch number | No | 2 | -0.50±2.121 | 0.870a | 1 | -1.00±0.00 | 0.471 |
|  | 1 | 2 | -6.50±38.891 |  | 3 | -1.33±19.140 |  |
|  | 2 | 6 | -5.33±8.140 |  | 8 | -8.37±5.528 |  |
|  | 3 | 30 | -6.70±12.540 |  | 28 | -12.64±16.081 |  |
|  | ≥4 | 4 | 0.01±14.259 |  | 3 | 0.33±21.548 |  |
| FB | No | 5 | -6.80±7.629 | 0.831 | 5 | -8.00±6.856 | 0.772 |
|  | Yes | 39 | -5.46±13.566 |  | 38 | -10.13±16.017 |  |
| HB | No | 8 | -4.75±16.175 | 0.838 | 10 | -7.70±10.924 | 0.61 |
|  | Yes | 36 | -5.81±12.416 |  | 33 | -10.55±16.359 |  |
| UB | No | 10 | -2.70±14.568 | 0.425 | 9 | -4.67±13.620 | 0.252 |
|  | Yes | 34 | -6.47±12.570 |  | 34 | -11.26±15.479 |  |
| ExB | No | 35 | -6.74±13.378 | 0.26 | 35 | -10.74±15.153 | 0.445 |
|  | Yes | 9 | -1.22±10.791 |  | 8 | -6.12±15.833 |  |
| IPDV drainage | Type I | 32 | -7.12±13.807 | 0.402 | 31 | -9.94±16.868 | 0.925 |
|  | Non-Type I | 15 | -3.73±10.222 |  | 16 | -10.37±10.327 |  |
| CIPV drainage | IMV | 22 | -6.05±6.373 | 0.066^a^ | **24** | **-14.00±15.698** | **0.025** |
|  | SMV | 5 | -3.00±9.055 |  | 6 | -3.00±3.847 |  |
|  | SpV | 2 | -20.00±19.799 |  | 2 | -10.00±12.728 |  |
|  | Colonic vein or JVT | 9 | 4.00±11.511 |  | 9 | 0.11±13.788 |  |
|  | Dual drainage pattern | 4 | -16.00±27.725 |  | **3** | **-27.00±10.536** |  |
| CIPV drainage | **IMV** | 24 | -8.08±12.076 | 0.084 | **25** | **-14.96±16.100** | **0.019** |
|  | Non-IMV | 18 | -1.22±12.850 |  | 19 | -4.21±12.109 |  |
| Postoperative fistula | No | 14 | -4.00±8.832 | 0.812 | 15 | -4.53±9.538 | 0.087 |
|  | Biochemical | 17 | -5.06±15.821 |  | 18 | -8.33±13.240 |  |
|  | B/C | 21 | -6.71±11.498 |  | 19 | -15.21±17.342 |  |
| Postoperative infection | No | 39 | -5.21±13.518 | 0.813 | 41 | -8.46±14.917 | 0.219 |
|  | Yes | 13 | -6.15±8.040 |  | 11 | -14.55±12.069 |  |
| Postoperative transfusion | No | 47 | -4.81±9.764 | 0.629 | 47 | -8.11±10.222 | 0.321 |
|  | Yes | 5 | -11.40±28.050 |  | 5 | -25.20±33.730 |  |
| Postoperative intervention | No | 46 | -6.07±12.828 | 0.317 | 47 | -7.79±11.436 | 0.159 |
|  | Yes | 6 | -0.67±5.820 |  | 5 | -28.20±26.357 |  |
| Length of stay | days | R=0.121,P=0.394 | | | R=-0.134,P=0.344 | | |

**Supplementary Table 7: The analysis of PPH and intraoperative bleeding-related factors in patients undergoing distal pancreatic surgery**

| DPA variation | | PPH | | | Intraoperative bleeding (mL) | | | ΔHb (POD1-Pre) (g/L) | | |
| --- | --- | --- | --- | --- | --- | --- | --- | --- | --- | --- |
|  |  | No | Yes | P | N | Mean±SD | P | N | Mean±SD | P |
| Gender | Female | 44 (50.6%) | 2 (100%) | 0.495 | 46 | 188.91±258.760 | 0.731 | 46 | -10.24±11.090 | 0.094 |
|  | Male | 43 (49.4%) | 0 (0%) |  | 43 | 173.02±162.195 |  | 42 | -6.55±9.179 |  |
| Age | years | 54.15±14.205 | 49.50±20.506 | 0.65 | R=0.110,P=0.305 | | | R=0.090,P=0.405 | | |
| Surgical method | Open | 1 (1.1%) | 0 (0%) | 0.844 | 1 | 800±0.00 | 0.206a | 1 | -6±0.0 | 0.969 |
|  | LAP | 27 (31%) | 1 (50%) |  | 28 | 197.14±307.762 |  | 27 | -8.37±11.839 |  |
|  | Robotic | 59 (67.8%) | 1 (50%) |  | 60 | 163.50±140.058 |  | 60 | -8.57±9.774 |  |
| Pathology type | Benign | 60 (69.0%) | 2 (100%) | 0.345 | 62 | 182.26±231.937 | 0.947 | 61 | -9.69±10.807 | 0.099 |
|  | Malignant | 27 (31.0%) | 0 (0%) |  | 27 | 178.89±179.943 |  | 27 | -5.74±8.747 |  |
| Origin type | IA | 19 (21.8%) | 0 (0%) | 0.308 | 19 | 265.26±353.025 | 0.658 | 19 | -9.21±9.992 | 0.331 |
|  | IB | 12 (13.8%) | 1 (50%) |  | 13 | 151.54±110.140 |  | 13 | -9.08±13.351 |  |
|  | IC | 7 (8%) | 1 (50%) |  | 8 | 151.25±93.570 |  | 8 | -16.75±11.145 |  |
|  | IIA | 23 (26.4%) | 0 (0%) |  | 23 | 155.22±189.374 |  | 23 | -6.83±9.861 |  |
|  | IIB | 12 (13.8%) | 0 (0%) |  | 12 | 180.83±174.952 |  | 11 | -7.09±8.018 |  |
|  | III | 5 (5.7%) | 0 (0%) |  | 5 | 206±148.930 |  | 5 | -7±8.155 |  |
|  | V | 9 (10.3%) | 0 (0%) |  | 9 | 126.67±180.555 |  | 9 | -5.44±9.315 |  |
| Branch number | No | 2 (2.6%) | 0 (0%) | 0.541 | 2 | 75±35.355 | 0.895a | 2 | -7±8.485 | 0.575 |
|  | 1 | 9 (11.5%) | 1 (50%) |  | 10 | 297±474.577 |  | 10 | -13.5±12.791 |  |
|  | 2 | 22 (28.2%) | 0 (0%) |  | 22 | 184.09±148.634 |  | 22 | -6.82±8.781 |  |
|  | 3 | 38 (48.7%) | 1 (50%) |  | 39 | 172.56±162.751 |  | 38 | -8.66±11.376 |  |
|  | ≥4 | 7 (9.0%) | 0 (0%) |  | 7 | 155.71±169.790 |  | 7 | -9.86±6.149 |  |
| FB | No | 8 (10.3%) | 0 (0%) | 0.633 | 8 | 201.25±181.379 | 0.852 | 8 | -10.37±10.391 | 0.660 |
|  | Yes | 70 (89.7%) | 2 (100%) |  | 72 | 185.83±225.206 |  | 71 | -8.65±10.505 |  |
| HB | No | 22 (28.2%) | 1 (50%) | 0.501 | 23 | 172.61±127.715 | 0.706 | 23 | -11.22±10.514 | 0.193 |
|  | Yes | 56 (71.8%) | 1 (50%) |  | 57 | 193.33±248.821 |  | 56 | -7.84±10.343 |  |
| UB | No | 19 (24.4%) | 1 (50%) | 0.408 | 20 | 143±116.488 | 0.301 | 20 | -5.3±10.373 | 0.081 |
|  | Yes | 59 (75.6%) | 1 (50%) |  | 60 | 202.17±244.347 |  | 59 | -10.02±10.276 |  |
| ExB | No | 71 (91.0%) | 2 (100%) | 0.657 | 73 | 191.78±228.037 | 0.567 | 72 | -9.11±10.569 | 0.435 |
|  | Yes | 7 (9.0%) | 0 (0%) |  | 7 | 141.43±110.065 |  | 7 | -5.86±9.155 |  |
| IPDV drainage | Type I | 1 (50.0%) | - | - | 1 | 300 | - | 1 | 3 | - |
|  | Non-Type I | 1 (50.0%) | - |  | 1 | 500 |  | 1 | -13 |  |
| CIPV drainage | IMV | 37 (49.3%) | 1 (50%) | 0.327 | 38 | 165±143.277 | 0.578a | 37 | -10.19±9.565 | **0.038**^a^ |
|  | SMV | 11 (14.7%) | 0 (0%) |  | 11 | 97.27±72.124 |  | 11 | -6.91±4.742 |  |
|  | SpV | 6 (8.0%) | 1 (50%) |  | 7 | 401.43±562.181 |  | 7 | -15.71±14.186 |  |
|  | Colonic vein or JVT | 14 (18.7%) | 0 (0%) |  | 14 | 158.57±124.521 |  | 14 | -5.79±12.217 |  |
|  | Dual drainage pattern | 7 (9.3%) | 0 (0%) |  | 7 | 172.86±132.378 |  | 7 | 0.86±7.010 |  |
| CIPV drainage | IMV | 32 (42.7%) | 1 (50%) | 0.836 | 44 | 163.18±140.328 | 0.534 | 43 | -8.56±10.091 | 0.877 |
|  | Non-IMV | 43 (57.3%) | 1 (50%) |  | 33 | 193.94±284.099 |  | 33 | -8.18±11.032 |  |
| Postoperative fistula | No | 3 (3.4%) | 0 (0%) | 0.951 | 3 | 210±253.574 | 0.819 | 3 | -6.33±18.230 | 0.699 |
|  | Biochemical | 37 (42.5%) | 1 (50%) |  | 38 | 195.79±292.821 |  | 37 | -7.57±8.140 |  |
|  | B/C | 47 (54.0%) | 1 (50%) |  | 48 | 167.92±130.742 |  | 48 | -9.31±11.449 |  |
| Postoperative infection | No | 82 (94.3%) | 2 (100%) | 0.727 | 84 | 182.14±221.147 | 0.872 | 83 | -8.33±10.316 | 0.577 |
|  | Yes | 5 (5.7%) | 0 (0%) |  | 5 | 166±125.618 |  | 5 | -11±11.489 |  |
| Postoperative transfusion | No | 87 (100%) | 1 (50%) | 0.022 | 88 | 181.02±217.70 | - | 87 | -8.10±9.775 | - |
|  | Yes | 0 (0%) | 1 (50%) |  | 1 | 200.00 |  | 1 | -41.00 |  |
| Postoperative intervention | No | 87 (100%) | 1 (50%) | 0.022 | 88 | 181.02±217.70 | - | 87 | -8.10±9.775 | - |
|  | Yes | 0 (0%) | 1 (50%) |  | 1 | 200.00 |  | 1 | -41.00 |  |
| Length of stay | days | 9.86±4.415 | 14.00±0.00 | 0.191 | R=0.044,P=0.685 | | | R=0.050,P=0.644 | | |

**Supplementary Table 8: The analysis of postoperative hemoglobin decline-related factors in patients undergoing distal pancreatic surgery**

| DPA variation | | ΔHb (POD2-POD1) (g/L) | | | ΔHb (POD3-POD1) (g/L) | | |
| --- | --- | --- | --- | --- | --- | --- | --- |
|  |  | N | Mean±SD | P | N | Mean±SD | P |
| Gender | Female | 29 | -5.17±6.703 | 0.942 | 45 | -10.67±7.628 | 0.992 |
|  | Male | 25 | -5.04±6.516 |  | 40 | -10.65±7.066 |  |
| Age | years | R=-0.049,P=0.724 | | | R=0.122,P=0.266 | | |
| Surgical method | Open | 1 | 0±0.0 | 0.205 | 1 | -15±0.0 | 0.653 |
|  | LAP | 18 | -7.22±6.025 |  | 26 | -11.46±8.377 |  |
|  | Robotic | 35 | -4.17±6.693 |  | 58 | -10.22±6.890 |  |
| Pathology type | Benign | 36 | -5.78±6.800 | 0.295 | 60 | -11.62±6.965 | 0.061 |
|  | Malignant | 18 | -3.78±5.996 |  | 25 | -8.36±7.794 |  |
| Origin type | IA | 11 | -7.91±7.355 | 0.442 | 18 | -12.94±6.673 | 0.436 |
|  | IB | 9 | -4±5.385 |  | 13 | -12.08±9.367 |  |
|  | IC | 7 | -1.43±9.235 |  | 7 | -7.14±9.477 |  |
|  | IIA | 11 | -4.73±5.867 |  | 22 | -9.68±6.027 |  |
|  | IIB | 7 | -7.71±7.158 |  | 11 | -8.55±7.673 |  |
|  | III | 3 | -3±3.00 |  | 5 | -9.2±3.962 |  |
|  | V | 6 | -4.67±3.670 |  | 9 | -12.56±7.282 |  |
| Branch number | No | 2 | -9.5±0.707 | 0.598 | 2 | -5±12.728 | 0.338 |
|  | 1 | 6 | -4.67±10.671 |  | 9 | -8.67±7.984 |  |
|  | 2 | 12 | -6.83±7.408 |  | 21 | -8.67±7.939 |  |
|  | 3 | 22 | -3.64±5.430 |  | 37 | -11.97±6.602 |  |
|  | ≥4 | 6 | -6.50±7.503 |  | 7 | -11.43±7.044 |  |
| FB | No | 6 | -7.17±5.776 | 0.451 | 7 | -8.57±7.997 | 0.485 |
|  | Yes | 42 | -4.88±7.009 |  | 69 | -10.62±7.313 |  |
| HB | No | 16 | -5.37±8.429 | 0.884 | 22 | -7.45±8.667 | **0.049** |
|  | Yes | 32 | -5.06±6.069 |  | 54 | -11.65±6.434 |  |
| UB | No | 10 | -6.8±7.436 | 0.403 | 19 | -9.47±7.756 | 0.514 |
|  | Yes | 38 | -4.74±6.733 |  | 57 | -10.75±7.246 |  |
| ExB | No | 41 | -4.73±6.753 | 0.292 | 69 | -10.06±7.135 | 0.162 |
|  | Yes | 7 | -7.71±7.432 |  | 7 | -14.14±8.934 |  |
| IPDV drainage | Type I | - | - | - | 1 | -23 | - |
|  | Non-Type I | 1 | -10 |  | 1 | -15 |  |
| CIPV drainage | IMV | 20 | -4.9±5.730 | 0.787 | 36 | -10.83±7.493 | 0.414 |
|  | SMV | 8 | -6.5±4.751 |  | 11 | -9.64±6.546 |  |
|  | SpV | 3 | -2.67±14.503 |  | 7 | -6.43±8.284 |  |
|  | Colonic vein or JVT | 11 | -3.73±7.773 |  | 13 | -9.23±6.978 |  |
|  | Dual drainage pattern | 4 | -7.5±3.873 |  | 7 | -13.57±6.079 |  |
| CIPV drainage | **IMV** | 24 | -5.33±5.483 | 0.705 | 42 | -11.07±7.246 | 0.247 |
|  | Non-IMV | 22 | -4.59±7.657 |  | 32 | -9.09±7.177 |  |
| Postoperative fistula | No | 2 | -12.5±2.121 | 0.227 | 3 | -10.67±3.055 | 0.394 |
|  | Biochemical | 23 | -5.43±6.402 |  | 35 | -9.37±7.893 |  |
|  | B/C | 29 | -4.34±6.662 |  | 47 | -11.62±7.023 |  |
| Postoperative infection | No | 51 | -5.24±6.581 | 0.571 | 80 | -10.84±7.454 | 0.372 |
|  | Yes | 3 | -3±7.0 |  | 5 | -7.8±4.324 |  |
| Postoperative transfusion | No | 53 | -5.43±6.169 | - | 84 | -10.89±7.042 | - |
|  | Yes | 1 | 12.00 |  | 1 | 9.00 |  |
| Postoperative intervention | No | 53 | -5.43±6.169 | - | 84 | -10.89±7.042 | - |
|  | Yes | 1 | 12.00 |  | 1 | 9.00 |  |
| Length of stay | days | R=0.167,P=0.227 | | | R=0.001,P=0.991 | | |

**Supplementary Table 9a: Multivariable analysis of three vessels associated with ΔHb_POD3-POD1**

|  |  |  | B with 95% CI | |  |
| --- | --- | --- | --- | --- | --- |
|  | B | Std. Error | Lower Bound | Upper Bound | Sig. P |
| DPA variations | -1.076 | 1.231 | -3.567 | 1.415 | 0.387 |
| IPDV drainage | 0.795 | 4.79 | -8.902 | 10.492 | 0.869 |
| CIPV drainage | -11.226 | 4.697 | -20.736 | -1.716 | **0.022** |

**Supplementary Table 9b: Adjusted multivariable analysis of three vessels associated with ΔHb_POD3-POD1**

|  |  |  | B with 95% CI | |  |
| --- | --- | --- | --- | --- | --- |
|  | B | Std. Error | Lower Bound | Upper Bound | Sig. P |
| DPA variations | -2.005 | 2.139 | -6.42 | 2.41 | 0.358 |
| IPDV drainage | 1.982 | 6.623 | -11.688 | 15.652 | 0.767 |
| CIPV drainage | -11.417 | 7.363 | -26.614 | 3.78 | 0.134 |
| Surgical method | 0.29 | 5.237 | -10.519 | 11.099 | 0.956 |
| Resection area | -0.33 | 18.792 | -39.114 | 38.455 | 0.986 |
| Pathology type | -7.468 | 7.336 | -22.608 | 7.672 | 0.319 |
| FB | -1.015 | 11.424 | -24.592 | 22.562 | 0.93 |
| HB | 1.046 | 7.815 | -15.085 | 17.176 | 0.895 |
| UB | -6.624 | 7.287 | -21.663 | 8.416 | 0.372 |
| ExB | -2.302 | 8.735 | -20.329 | 15.725 | 0.794 |

**Supplementary Table 10: The DPA classification, clinical intervention and transfusion of patients with PPH**

| No. | Surgery | | DPA origin | | Intraoperative bleeding  (mL) | Intraoperative transfusion  (RBC) | PPH grade | Postoperative RBC transfusion prior to 7:00 AM, POD2 | **Clinical intervention** |
| --- | --- | --- | --- | --- | --- | --- | --- | --- | --- |
| PUMC_DPA009 | PD | Open | CA | IC | 2500 | 4 U | B | NO | ICU care, total transfusion: 800 mL plasma + 4U RBC; CT-guided percutaneous drainage |
| PUMC_DPA028 | PD | Robotic | SpA | IA | 400 | 0 U | C | YES | Gastrointestinal bleeding, total transfusion: 6U RBC; enhanced CT + CTA performed; nasogastric tube placed |
| PUMC_DPA033 | Warshaw | Robotic | CA | IC | 200 | 0 U | B | YES | Intra-abdominal bleeding, total transfusion: 3 U RBC; interventional angiography showed no active bleeding |
| PUMC_DPA039 | PD | LAP | aRHA | III | 1000 | 2 U | C | NO | Intra-abdominal bleeding; abdominal enhanced CTA revealed active bleeding; interventional embolization performed (bleeding from small branch of right hepatic artery origin); transfusion + ICU care |
| PUMC_DPA041 | PD | Robotic | SpA | IA | 2000 | 4 U | B | YES | Significant hemoglobin drop; transfusion given (2U RBC + 800 mL plasma) |
| PUMC_DPA159 | TP | Robotic | SpA | IA | 1100 | 2 U | B | NO | Postoperative anemia, delayed extraluminal bleeding; multiple transfusions required |
