# Supplementary material for: Impact of precise preoperative vascular assessment and different dorsal pancreatic artery variant subtypes on pancreatic surgery-related bleeding
Source: BMC Gastroenterol. 2026 Feb 14;26:186. doi: 10.1186/s12876-026-04687-8 (PMC13011292; doi:10.1186/s12876-026-04687-8)
Supplement: Supplementary file 3 — Supplementary Material 3. [file 12876_2026_4687_MOESM3_ESM.docx]

**Supplementary Figure 1: Study procedures.** a. Patients that were not preoperatively evaluated by using PAAF-PVV (non-precise assessment) were retrospectively included at Peking Union Medical College Hospital (PUMCH) between January 2022 and September 2022. b. Patients that were preoperatively evaluated by using PAAF-PVV (precise assessment) were prospectively included at PUMCH between December 2022 and April 2024.


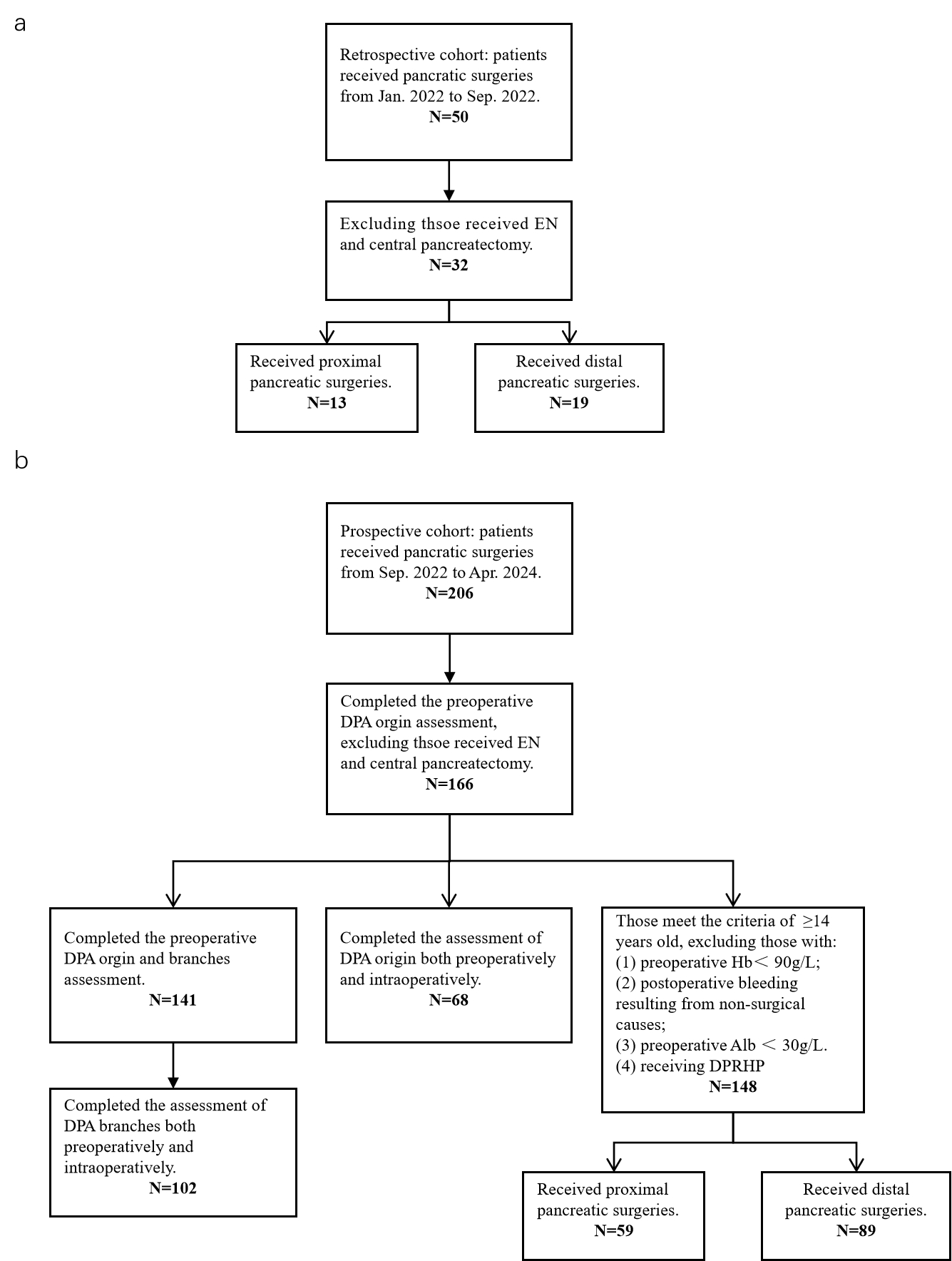


**Supplementary Figure 2: Subgroup analysis between Non-precise assessment and precise assessment group.** a. Intraoperative bleeding volume (mL); b. ΔHb_POD1-pre （g/L）; c. ΔHb_POD3-POD1 （g/L）; d. ΔHb_POD2-POD1 （g/L）; e. Intraoperative bleeding volume (mL); f. ΔHb_POD1-pre （g/L）; g. ΔHb_POD3-POD1 （g/L）. Non-Precise: patients receiving non-precise preoperative vascular assessment (n=32); Precise: patients receiving precise preoperative vascular assessment (n=148). The sub-group analysis in a, b, c was not adjusted for covariates. The sub-group analysis in d, e, f, g was adjusted for covariates. These forest plots present exploratory subgroup analyses, indicating the preliminary nature of the findings.

a.


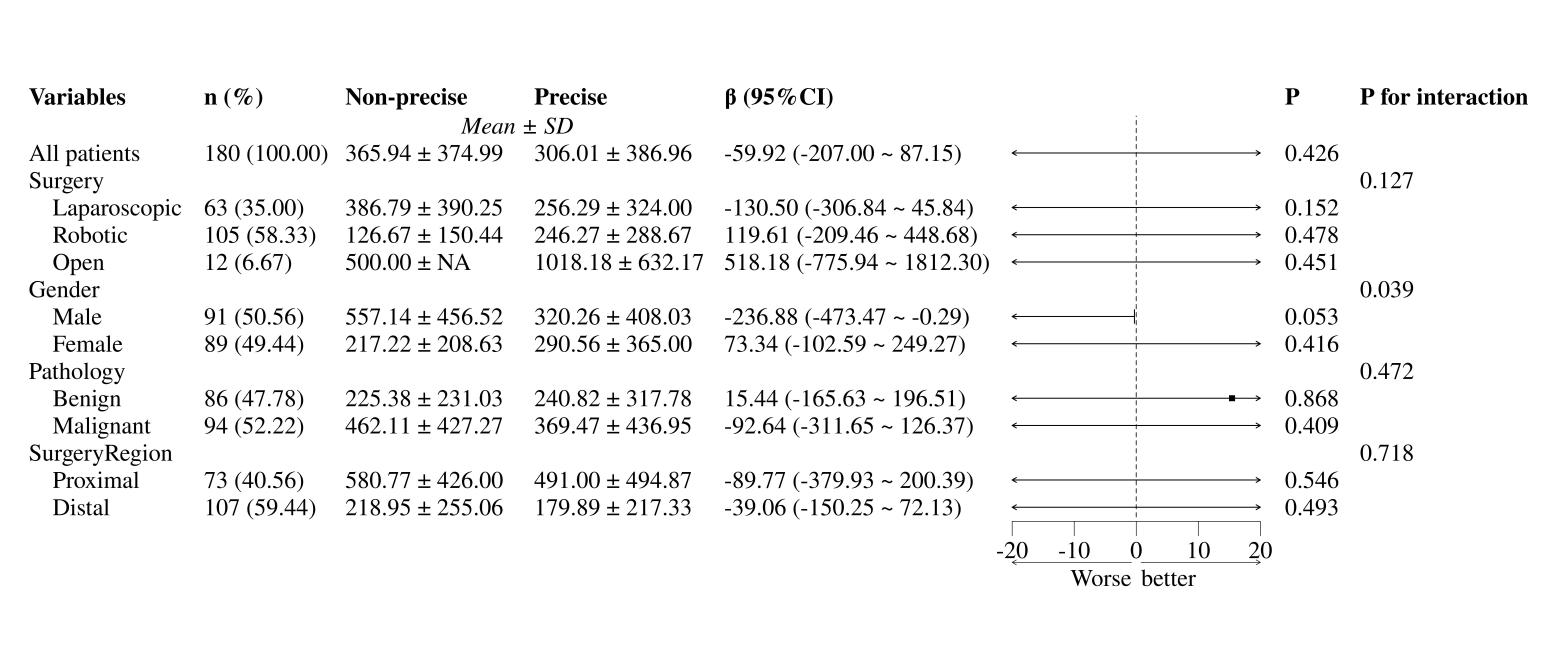


b.


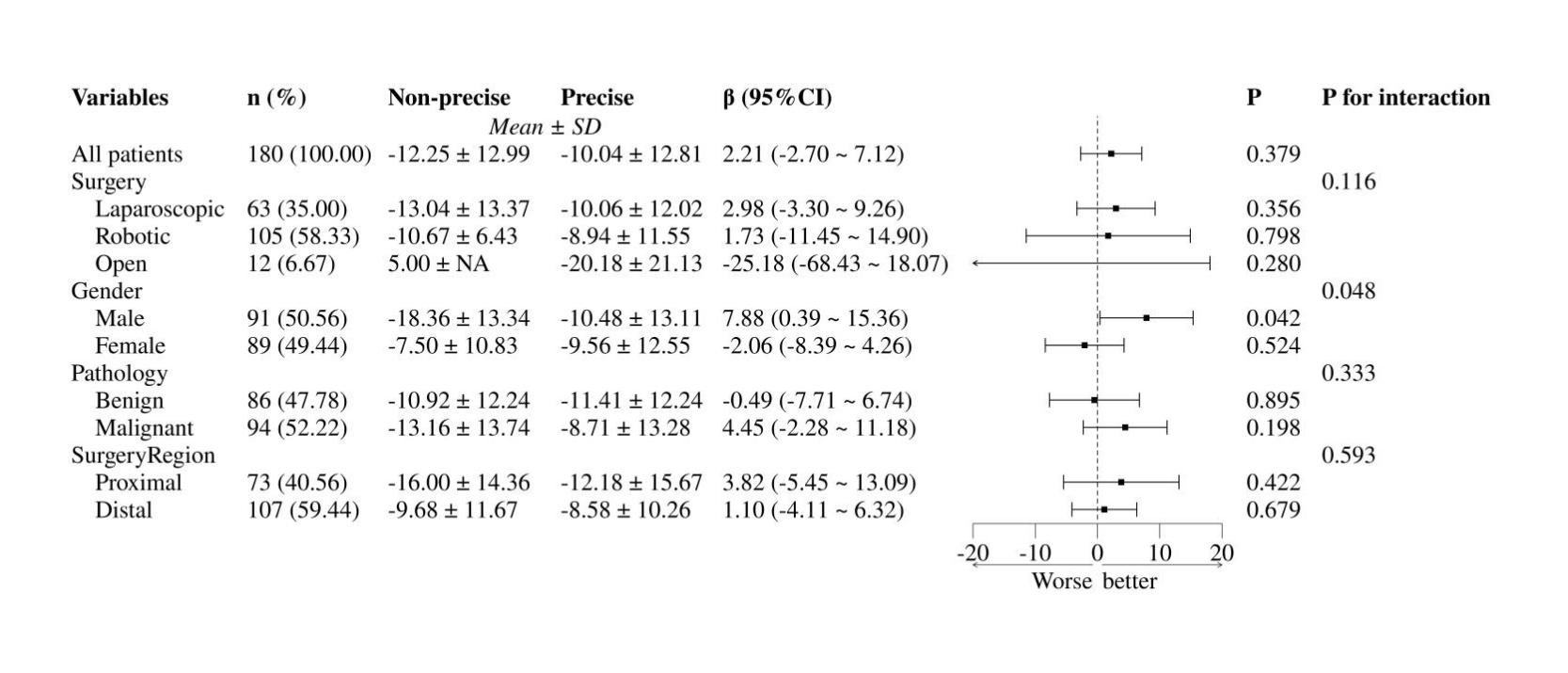


c.


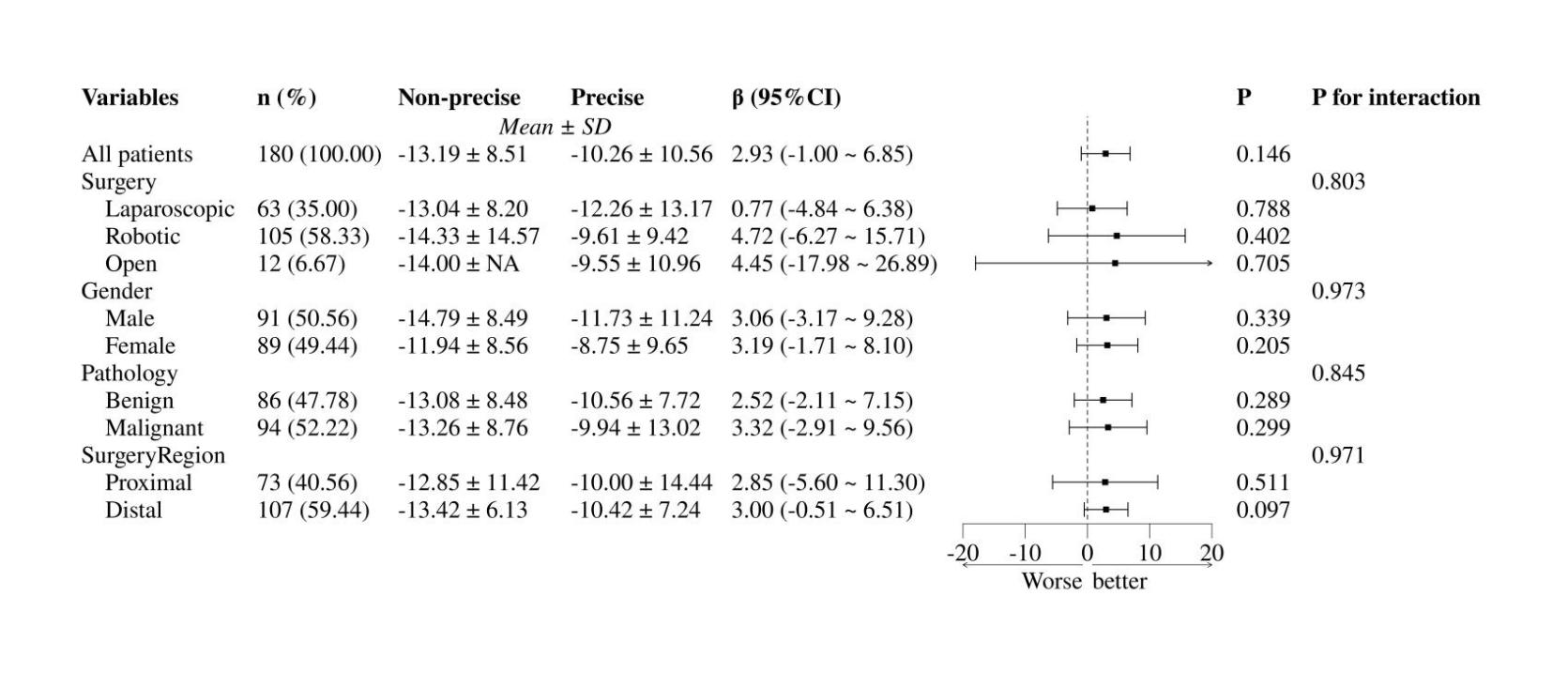


d.


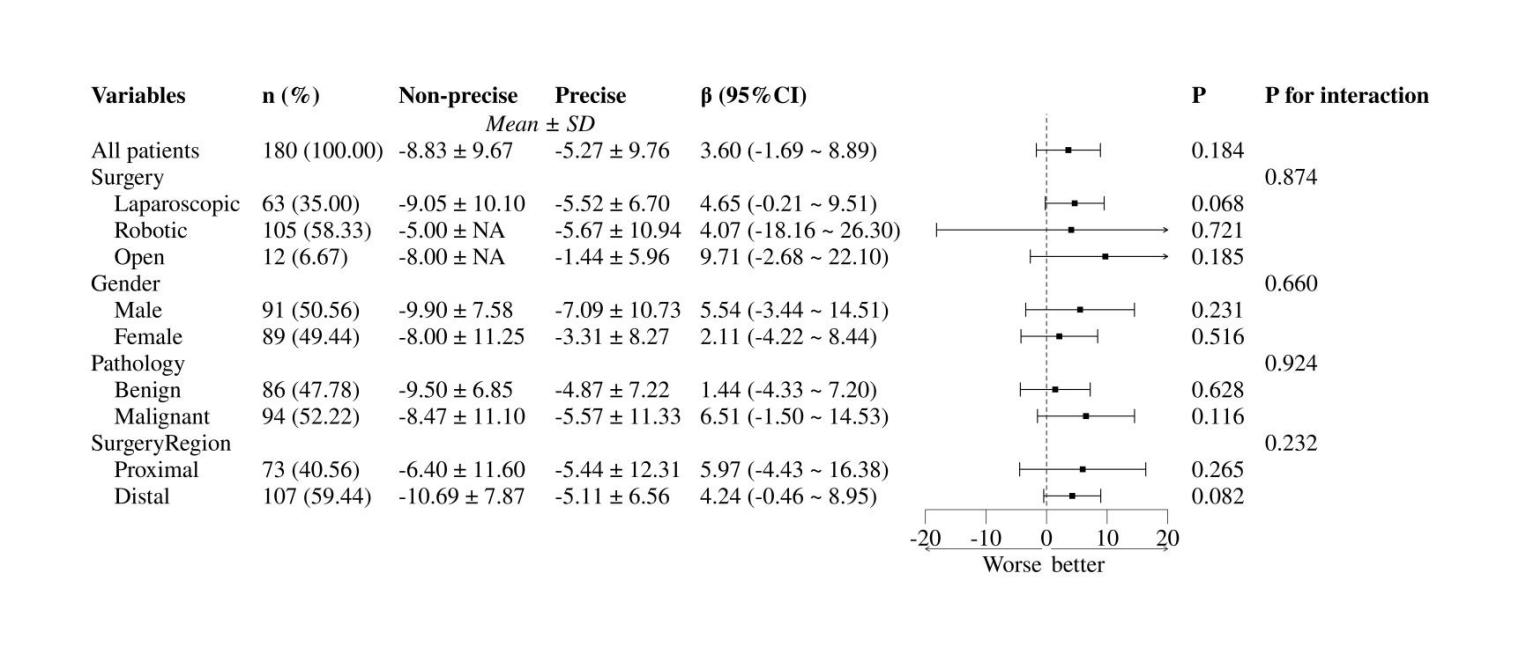


e.


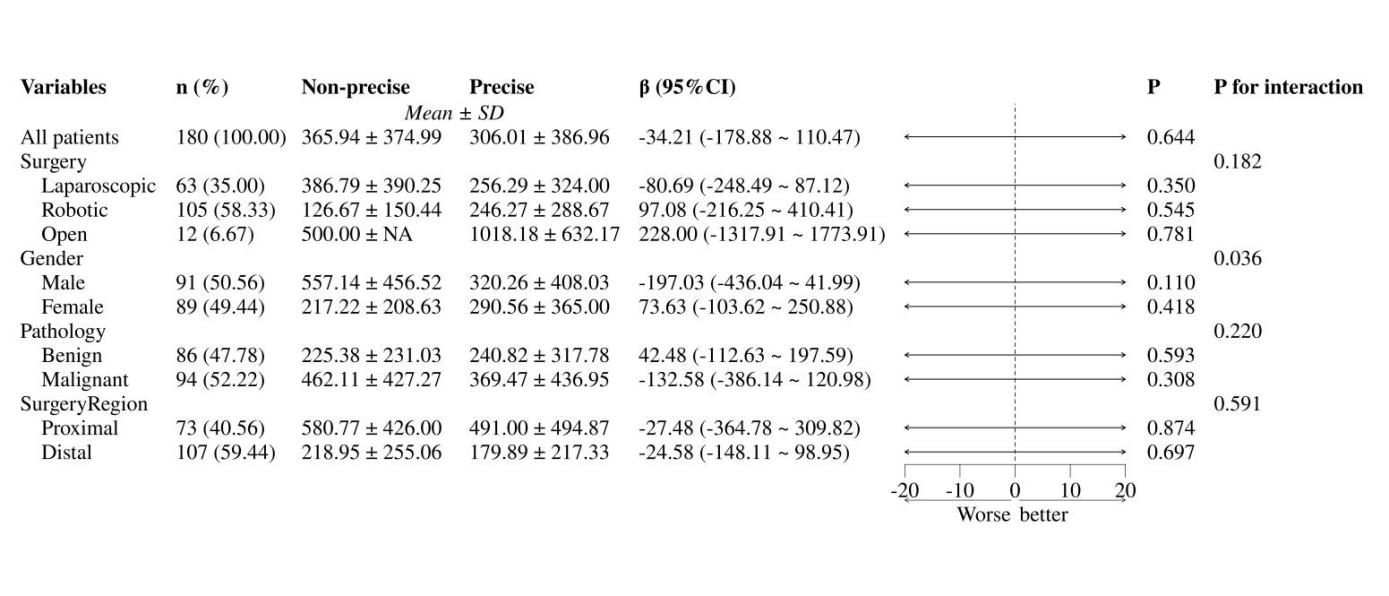


f.


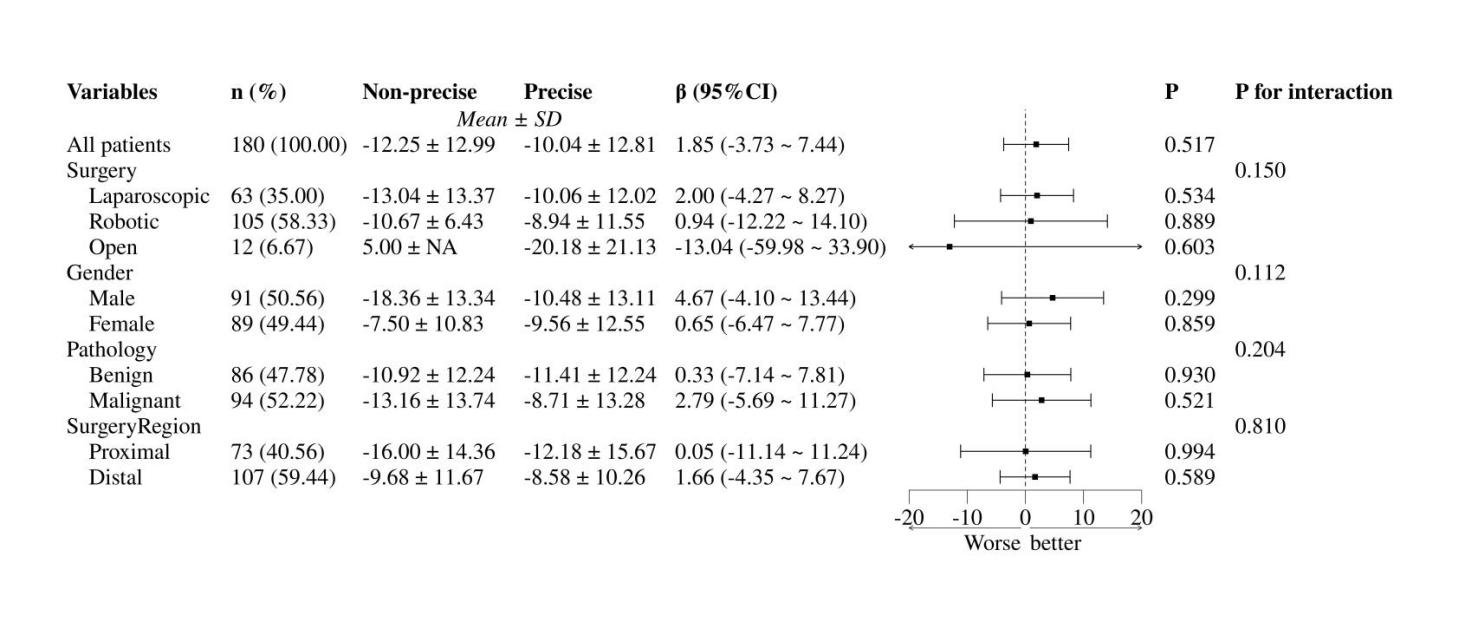


g.


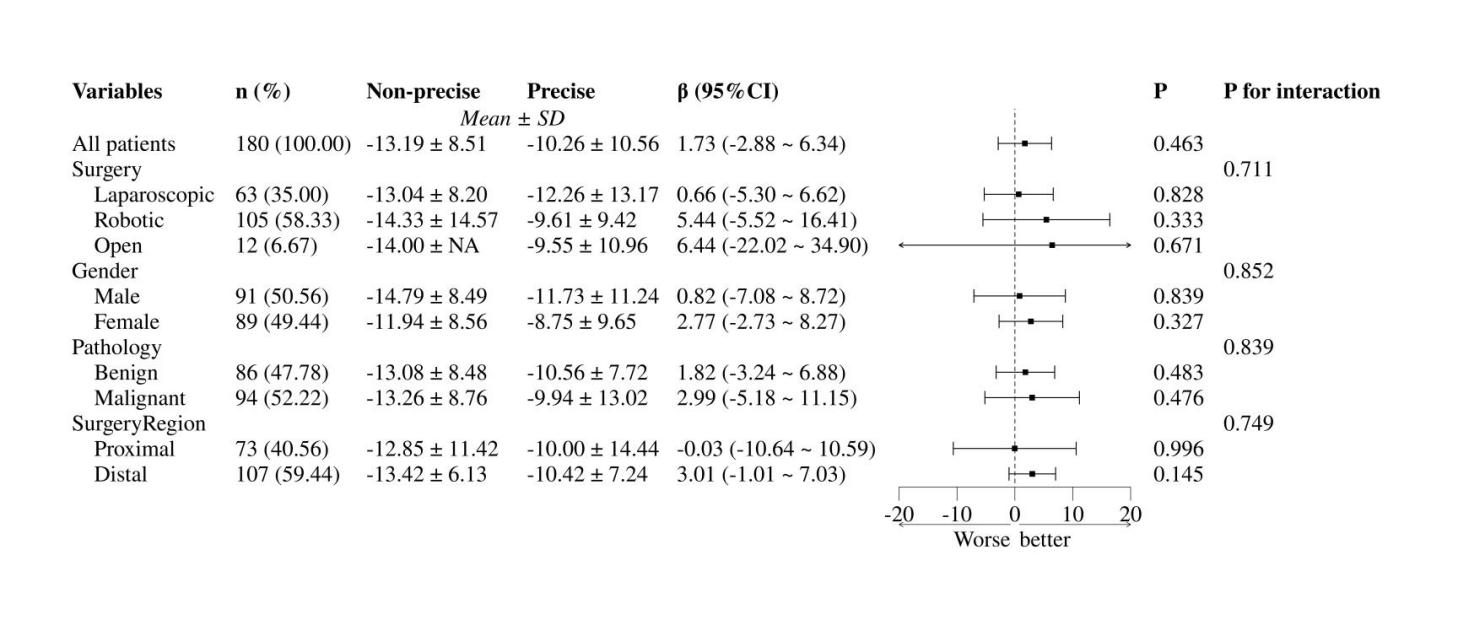


**Supplementary Figure 3 Type IA DPA diagram.** a-b The 3D virtual and zoomed-in images showing the origin and branches of type IA DPA that originates from SpA and has the branches of HB, UB and FB. c-e The 3D printing model and zoomed-in images showing the origin and branches of type IA DPA that was showed in a-b. DPA is marked by black thread.

Abbreviations: SMA superior mesenteric artery; SMV superior mesenteric vein; CA celiac trunk; DPA dorsal pancreatic artery; SpA splenic artery; HB head-side branch; UB Uncinate process branch; FB foot-side branch.

**Supplementary Figure 4 Type IB DPA diagram.** a-b The 3D virtual and zoomed-in images showing the origin and branches of type IB DPA that originates from CHA and has the branches of HB, UB and FB. c-e The 3D printing model and zoomed-in images showing the origin and branches of type IB DPA that was showed in a-b. DPA is marked by black thread.

Abbreviations: CHA common hepatic artery; CA celiac trunk; DPA dorsal pancreatic artery; SpA splenic artery; HB head-side branch; UB Uncinate process branch; FB foot-side branch.

**Supplementary Figure 5 Type IIA DPA diagram.** a-b The 3D virtual and zoomed-in images showing the origin and branches of type IIA DPA that originates from SMA and has the branches of HB1, HB2, and UB. c-e The 3D printing model and zoomed-in images showing the origin and branches of type IIA DPA that was showed in a-b. DPA is marked by black thread.

Abbreviations: SMA superior mesenteric artery; SMV superior mesenteric vein; CA celiac trunk; DPA dorsal pancreatic artery; SpA splenic artery; HB head-side branch; UB Uncinate process branch; FB foot-side branch; IPDA inferior pancreaticoduodenal artery; JA jejunal artery.

**Supplementary Figure 6 Type IIB DPA diagram.** a-b The 3D virtual and zoomed-in images showing the origin and branches of type IIB DPA that originates from CMA. The CMA originates from SMA. c-e The 3D printing model and zoomed-in images showing the origin and branches of type IIB DPA that was showed in a-b. DPA is marked by black thread.

Abbreviations: SMA superior mesenteric artery; DPA dorsal pancreatic artery; SpA splenic artery; HB head-side branch; UB Uncinate process branch; FB foot-side branch; IPDA inferior pancreaticoduodenal artery; CMA referred to MCA, middle colonic artery.

**Supplementary Figure 7 Type III DPA diagram.** a-b The 3D virtual and zoomed-in images showing the origin and branches of type III DPA that originates from aRHA and has the branches of HB and FB. c-e The 3D printing model and zoomed-in images showing the origin and branches of type III DPA that was showed in a-b. DPA is marked by black thread.

Abbreviations: SMA superior mesenteric artery; DPA dorsal pancreatic artery; SpA splenic artery; HB head-side branch; UB Uncinate process branch; FB foot-side branch; aRHA aberrant right hepatic artery.
